# Structural basis for Fullerene geometry in a human endogenous retrovirus capsid

**DOI:** 10.1038/s41467-019-13786-y

**Published:** 2019-12-20

**Authors:** Oliver Acton, Tim Grant, Giuseppe Nicastro, Neil J. Ball, David C. Goldstone, Laura E. Robertson, Kasim Sader, Andrea Nans, Andres Ramos, Jonathan P. Stoye, Ian A. Taylor, Peter B. Rosenthal

**Affiliations:** 1 Structural Biology of Cells and Viruses Laboratory, The Francis Crick Institute, 1 Midland Road, London, NW1 1AT UK; 2 Macromolecular Structure Laboratory, The Francis Crick Institute, 1 Midland Road, London, NW1 1AT UK; 3 Division of Molecular Structure, MRC National Institute for Medical Research, London, NW7 1AA UK; 4 Retrovirus-Host Interactions Laboratory, The Francis Crick Institute, 1 Midland Road, London, NW1 1AT UK; 5 Department of Medicine, Imperial College London, London, SW7 2AZ UK; 6 Present Address: Department of Biochemistry, University of Washington, Seattle, WA USA; 7 Present Address: Howard Hughes Medical Institute, Janelia Research Campus, Ashburn, VA 20147 USA; 8 Present Address: School of Biological Sciences, University of Auckland, Auckland, New Zealand; 9 Present Address: Thermo Fisher Scientific Materials and Structural Analysis, Eindhoven, Netherlands; 10 Present Address: Institute of Structural and Molecular Biology, University College London, London, UK

**Keywords:** Cryoelectron microscopy, Solution-state NMR, X-ray crystallography, Viral proteins

## Abstract

The HML2 (HERV-K) group constitutes the most recently acquired family of human endogenous retroviruses, with many proviruses less than one million years old. Many maintain intact open reading frames and provirus expression together with HML2 particle formation are observed in early stage human embryo development and are associated with pluripotency as well as inflammatory disease, cancers and HIV-1 infection. Here, we reconstruct the core structural protein (CA) of an HML2 retrovirus, assemble particles *in vitro* and employ single particle cryogenic electron microscopy (cryo-EM) to determine structures of four classes of CA Fullerene shell assemblies. These icosahedral and capsular assemblies reveal at high-resolution the molecular interactions that allow CA to form both pentamers and hexamers and show how invariant pentamers and structurally plastic hexamers associate to form the unique polyhedral structures found in retroviral cores.

## Abstract (web summary)

In retroviruses, the capsid protein (CA) forms a shell surrounding the viral core. Here the authors combine cryo-electron microscopy with NMR and X-ray crystallography to examine the CA structure from the human endogenous retrovirus HML2 (HERV-K) and determine the structures of four Fullerene CA closed shells that reveal the molecular basis of capsid assembly.

## Introduction

The human genome contains a large number of endogenous retroviruses (ERVs) that provide a fossil record of human–pathogen interactions over millions of years^1,2^. In most instances, ERV open reading frames (ORFs) are degraded, containing stop codons, deletions and rearrangements. However, several members of the HML2 group of ERVs have proviruses less than one million years old^3–6^ with intact ORFs^7^ and HML2 particle formation is observed in early-stage human embryo development^8^ and is associated with pluripotency^9^, as well as inflammatory disease^10–12^, cancers^13–16^ and HIV-1 infection^17–19^. Moreover, viruses created by reconstruction of HML2 consensus sequences have been shown to produce infectious particles^20,21^.

Among the circulating exogenous retroviruses, the Gag polyprotein is processed into the matrix (MA), capsid (CA) and nucleocapsid (NC) proteins that form the structural layers within a mature retroviral particle. CA forms the capsid shell surrounding the viral core that protects and transports the viral genome and also interacts with host cell factors that are necessary for trafficking, nuclear entry and proviral integration^22–24^. Given these essential functions of the capsid, several structural studies have been undertaken that have provided the molecular details of the capsid’s individual hexameric and pentameric CA building blocks^25–28^. In addition, electron cryomicroscopy studies of whole retroviral cores and in vitro assemblies have provided insight into capsid assembly at low and medium resolution^29–34^. However, despite these advances the pleomorphic nature of retroviral capsids has so far confounded attempts to determine high-resolution structures of entire closed capsid shells containing both hexameric and pentameric subunits.

Here we express and purify CA (HML2 CA^rec^) from a consensus HML2 Gag ERV^20,21^ and determine crystal and solution nuclear magnetic resonance (NMR) structures of the N-terminal (CA^rec^-NTD) and C-terminal (CA^rec^-NTD) domains, respectively. Further, we assemble particles from HML2 CA^rec^ in vitro and use single-particle cryogenic electron microscopy (cryo-EM) to determine the high-resolution structures of four different types of Fullerene shell assemblies. Our data reveal that the structures of the (CA^rec^-NTD) and C-terminal (CA^rec^-NTD) domains are largely conserved with those of CA from exogenous retroviruses. Analysis of the shell structures reveals the intra- and inter-molecular interactions that drives CA assembly into pentamers and hexamers and their association into shells that encapsidate the retroviral genome.

## Results

### In vitro assembly and cryo-EM structures of HML2 CA shells

**Fig. 1:**
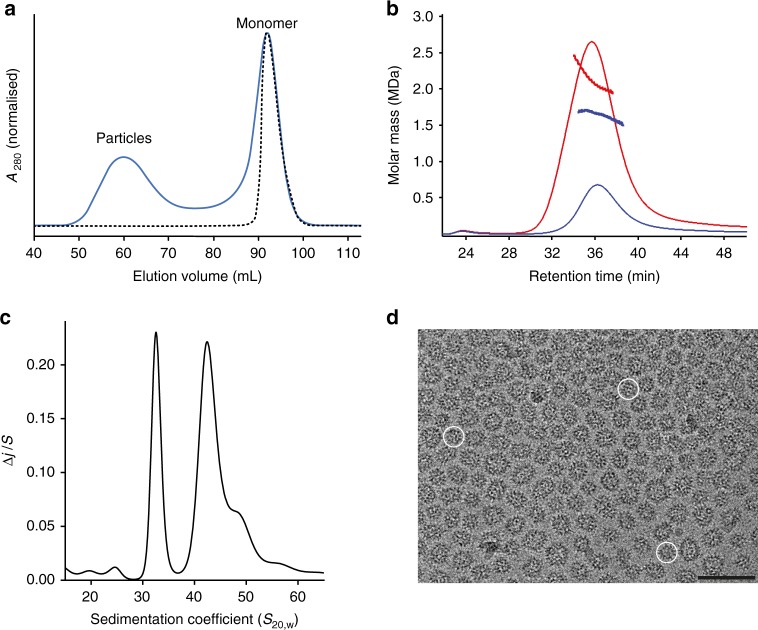
Hydrodynamic and EM analysis of HML2 CA^rec^ particles. **a** Gel filtration chromatogram (*A*_280_) of an HML2 CA^rec^ assembly reaction (1.4 M NaCl; solid blue line) and the unassembled monomer (0.1 M NaCl; dashed black line) separated on a Superose 6 16/60 GL column. The peaks containing HML2 CA^rec^ monomer and early eluting assembled particles are labelled. **b** Solution molecular mass of HML2 CA^rec^ particles. The molar mass distribution of HML2 CA^rec^ particles analysed by SEC-MALLS employing a Superose 6 10/30 column equilibrated in 1 M NaCl. The chromatograms were recorded from the 90° detector of the photometer from samples of HML2 CA^rec^ assembly reactions carried out at 2 mg mL^−1^ (blue) and 10 mg mL^−1^ (red). The points, colour-coded the same as the chromatograms, are the molar mass measured at 1-s intervals throughout the elution of the chromatographic peaks and show the presence of heterogeneous species ranging from 1.5 to 3.0 MDa. **c** Continuous distribution function of sedimentation coefficients. The plot shows the 20–60S region derived from sedimentation velocity analysis of a HML2 CA^rec^ particle preparation. HML2 CA^rec^ particles with sedimentation coefficients of 32S, 42S and 50S are the major species. **d** Cryo-electron micrograph of a field of HML2 CA^rec^ particles showing a predominance of small (T = 1) particles (circled examples) and larger capsular particles, scale bar is 50 nm.

To assess if human ERVs have retained the capacity to assemble CA into shells, we synthesised a codon-optimised consensus HML2 CA coding sequence^20,21^ (Supplementary Fig. 1) and expressed the protein, HML2 CA^rec^, in *Escherichia coli*. The addition of high salt (>1 M NaCl) to purified HML2 CA^rec^ induced the formation of high-molecular mass particles (Fig. 1a) and a strong concentration dependency of the size distribution (1.5–2.5 MDa) measured by size-exclusion chromatography (SEC) coupled to multi-angle laser light scattering (SEC-MALLS) demonstrated the presence of a heterogeneous mixture (Fig. 1b). Further resolution of the assembly reaction using sedimentation velocity revealed major 32S and 42S species together with a minor 50S component (Fig. 1c). Analysis by cryo-EM revealed a distribution of regular particles, comprising a large proportion of small 160 Å diameter spherical particles and additional larger capsular particles of up to 300 Å in the largest dimension (Fig. 1d).

**Fig. 2:**
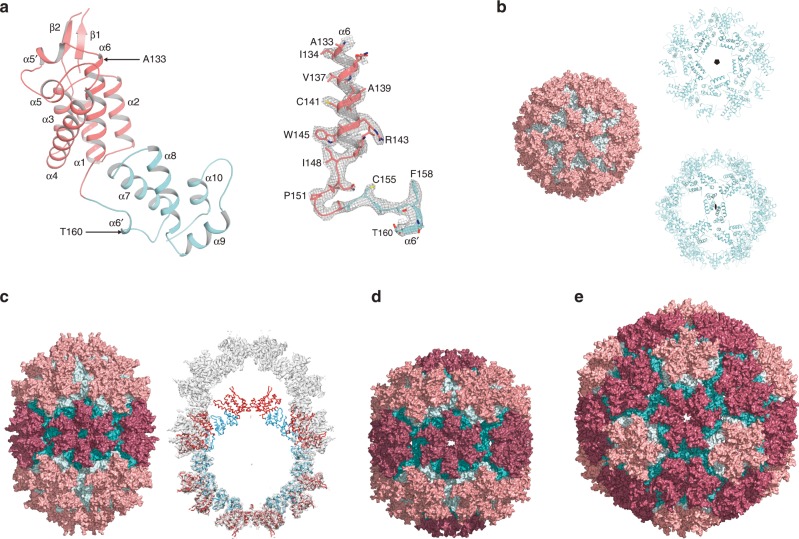
Structure of HML2 CA^rec^ monomer and assembled particles. **a** (Left) Cartoon representation of the HML2 CA^rec^ monomer from the T = 1 particle. The CA^rec^-NTD, residues P1 to S153, and CA^rec^-CTD, residues A154 to Q246, are coloured in pink and cyan, respectively. (Right) Experimental electron potential map (grey mesh) and model for the region indicated between α6 and α6′. Residues G152 to P156 in the linker region were built de novo into the density. **b** (Left) Surface representation of the T = 1 particle (60 monomers arranged as 12 pentamers) viewed along the five-fold symmetry axis with the NTDs and CTDs coloured as in **a**. (Right) The inner CTD “cage” viewed along the five-fold (upper) and the two-fold (lower). **c** (Left) D5 particle (90 monomers arranged as 12 pentamers and 5 hexamers) viewed along an equatorial pseudo-hexamer at the two-fold symmetry axis. Pentamer NTDs and CTDs are coloured as in **a**, and hexamer NTDs and CTDs are coloured in red and teal, respectively. (Right) Central slab of the D5 density map (grey surface) with the T = 1 particle aligned on the D5 polar pentamer. **d** D6 particle (108 monomers arranged as 12 pentamers and 8 hexamers) viewed along an equatorial pseudo-hexamer at the two-fold symmetry axis. Pentamers and hexamers are coloured as in **c**. **e** T = 3 particle (180 monomers arranged as 12 pentamers and 20 hexamers) viewed along an icosahedral two-fold symmetry axis. Pentamers and hexamers are coloured as in **c**.

Cryo-EM combined with single-particle image reconstruction was employed to determine the structure of these HML2 CA^rec^ particles. After two- (2D) and three-dimensional (3D) classification of particle images, the structures of four types of particle were resolved to high-resolution (Fig. 2 and Supplementary Table 1). Details of classification, image processing and map resolution are presented in (Supplementary Figs. 2–5). The resolution of the maps varies from 2.7 to 4.3 Å, but show clear density for backbone and side chains and are of sufficient quality for de novo building of atomic models (Fig. 2a and Supplementary Fig. 6). The majority species comprises 12 HML2 CA^rec^ pentamers with all interfaces equivalent, obeying perfect T = 1 icosahedral symmetry (Fig. 2b). The structure is resolved at the highest resolution (2.7 Å), likely corresponding to the 32S species observed in sedimentation experiments. In addition, there are a smaller number of D5 and D6 symmetric capsular structures (Fig. 2c, d) and a T = 3 icosahedral particle (Fig. 2e) that together likely constitute the faster sedimenting 42–46S species. The D5 particle is an elongated or capsular expansion of the T = 1 particle with polar regions identical to T = 1, but containing five equatorially inserted distorted hexamers, consistent with local two-fold (dihedral) symmetry (Fig. 2c). The D6 particles are also capsular with fully six-fold symmetric hexamers at the poles each surrounded by a ring of six pentamers and with six equatorial hexamers with similar distortions to those observed in the D5 structure (Fig. 2d). The largest structure is a T = 3 particle (Fig. 2e), resolved to 4.3 Å and contains a T = 1 particle within the interior, although no fixed orientation between the shells is apparent (Supplementary Fig. 5a, b). The T = 3 shell is 300 Å in diameter and is comprised of 12 pentamers, each surrounded by five hexamers. Hexamers are located at icosahedral three-fold axes and interact across icosahedral two-fold axes. This combination of the four structures describe, at high resolution, the molecular interactions within CA pentamer and hexamer arrangements, as well as the inter-pentamer, pentamer–hexamer and inter-hexamer interactions that participate in the formation of closed Fullerene shell structures in vivo.

To guide the structural interpretation of these particles at higher resolution, we determined the 1.8 Å crystal structure of the CA^rec^ N-terminal domain (CA^rec^-NTD) (Supplementary Fig. 7a and Supplementary Table 2) and the solution NMR structure of CA^rec^ C-terminal domain (CA^rec^-CTD) (Supplementary Fig. 7b, c and Supplementary Table 3). CA^rec^-NTD has the same N-terminal β-hairpin and six-helix fold observed in exogenous orthoretroviral CA proteins. CA^rec^-CTD comprises the same four-helix fold found in exogenous orthoretroviral CA-CTDs, but with an additional C-terminal distal helix (α11, residues A237-E248) that associates with the core of the CTD domain. Importantly, although α11 displays the sequential and medium-range inter-residue nuclear Overhauser effect (NOE) cross-peaks (Supplementary Fig. 7d) that define a helical conformation, the higher T_2_, lower T_1_ and lower heteronuclear ^1^H-^15^N NOE values observed in NMR relaxation experiments (Supplementary Fig. 8) together indicate it is more dynamic than the core domain (α7–α10). These high-resolution structures could be docked directly into the cryo-EM density and, with the additional building of the NTD–CTD linkers, the NTD–CTD interface and helix α8 at the CTD–CTD interface (Supplementary Fig. 6), refined well into each particle structure. Exceptions were the N-terminal β-hairpin that contains a large loop insertion and is more mobile than in the crystal structure, and the NTD–CTD linker region of D5 and D6 equatorial hexamers that displayed weaker density. In addition, the CTD α11 was not visible in these structures of mature particles, but given its proximity to the C terminus of CA, it may have a structural role in immature Gag assembly, similar to that observed for the SP1 spacer of HIV-1^35^ and CAH helix of murine leukaemia virus (MLV) Gag^32^.

Structural superimposition with exogenous retroviral CA domains revealed the strongest similarity of CA^rec^-NTD with the CA-NTD of the beta and alpharetroviruses Jaagsiekte sheep retrovirus (JSRV) and Rous sarcoma virus (RSV) (*Z*-scores 10.7 and 9.0) and the most distant relationship to the CA-NTD of the gammaretrovirus MLV (Supplementary Fig. 9a). Similarly, superimposition of HML2 CA^rec^-CTD with orthoretroviral CA-CTDs (Supplementary Fig. 9b) revealed comparable *Z*-scores for all available structures (7.4–5.9), except for the MLV CA-CTD that aligned significantly more poorly (*Z* = 5.0). These data agree with phylogenetic schemes placing HML2 close to an alpha and betaretroviral ancestor^36^ and also reveal that extant beta or alpharetroviruses, although none currently infect humans, have maintained the structural features of CA endogenized into the human genome.

### CA conformational switching in pentamers and hexamers

**Fig. 3:**
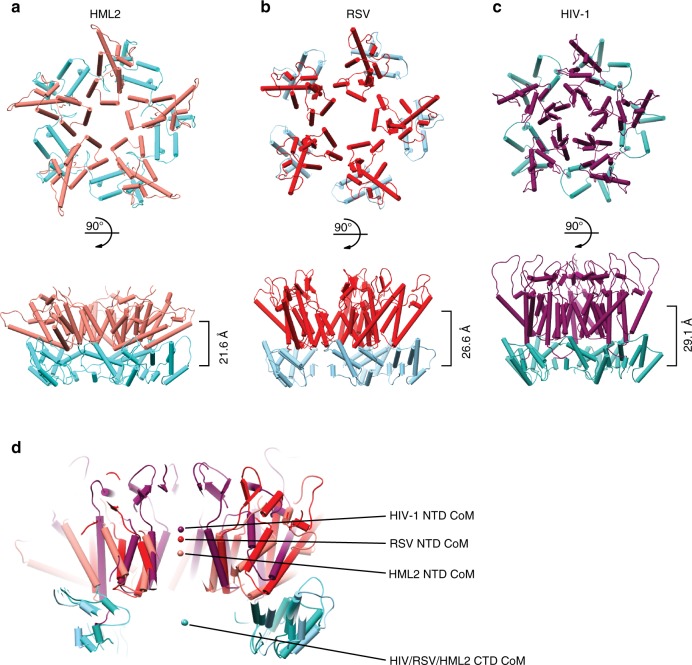
Comparison of HIV-1, RSV and HML2 CA pentamers. **a**–**c** Top and side view cartoon representations of CA pentamers with α-helices shown as cylinders, from **a** HML2 CA^rec^, **b** RSV (built from PDB: 1EM9 and 3G1I docked into EMD 5772) and **c** HIV-1 (PDB: 5MCY). NTDs are coloured pink, red and purple and CTDs are coloured light cyan, light blue and cyan for HML2, RSV and HIV-1, respectively. Pentamers are aligned with respect to their CTDs. In the lower panels, the distance between the centre of mass (CoM) for each NTD and CTD ring is shown on the right. **d** Central section through CA pentamers aligned with respect to CTDs. The CoM for NTD and CTD rings is represented by the spheres, colour coded as in **a**–**c** and shows the coincident CoM for CTDs and the difference in CTD–NTD vertical displacement for each pentamer.

The T = 1 particle comprises 12 HML2 CA^rec^ pentamers. The NTDs of each pentamer are associated proximal to the five-fold axis and form a layer of structure at a radius of 86.3 Å (NTD centroid distance) from the particle centre. Beneath the NTDs, the CTDs form an “inner cage” layer (Fig. 2b) at a radius of 64.7 Å (CTD centroid distance), and more distal to the five-fold axis. The NTD layer does not contribute to inter-pentamer interactions and so the entire inter-pentamer interface is mediated through inner cage CTD–CTD interactions across icosahedral two-fold axes (Fig. 2b). Comparison of the HML2 CA^rec^ pentamer to CA pentamers studied at lower resolution for HIV^29^ and RSV^33,37^ shows a good alignment of CTDs, that they occupy similar radial positions and employ the same inner cage CTD–CTD interactions at icosahedral two-fold axes. However, the relationship between the NTD and CTD layers in the different retroviral genera varies substantially (Fig. 3). In HML2, the vertical displacement between the NTD and CTD, as judged by the centre of mass (CoM) of the two layers, is 21.6 Å. In the RSV pentamer, this increases to 26.6 Å, and in the HIV-1 pentamer, the layers are even further apart at 29.1 Å (Fig. 3a–d). Therefore, although the underlying CTD sub-lattice and individual domain structures (Supplementary Fig. 9) are conserved across retroviral genera, the differences in the NTD–CTD posture appears to be genera specific.

**Fig. 4:**
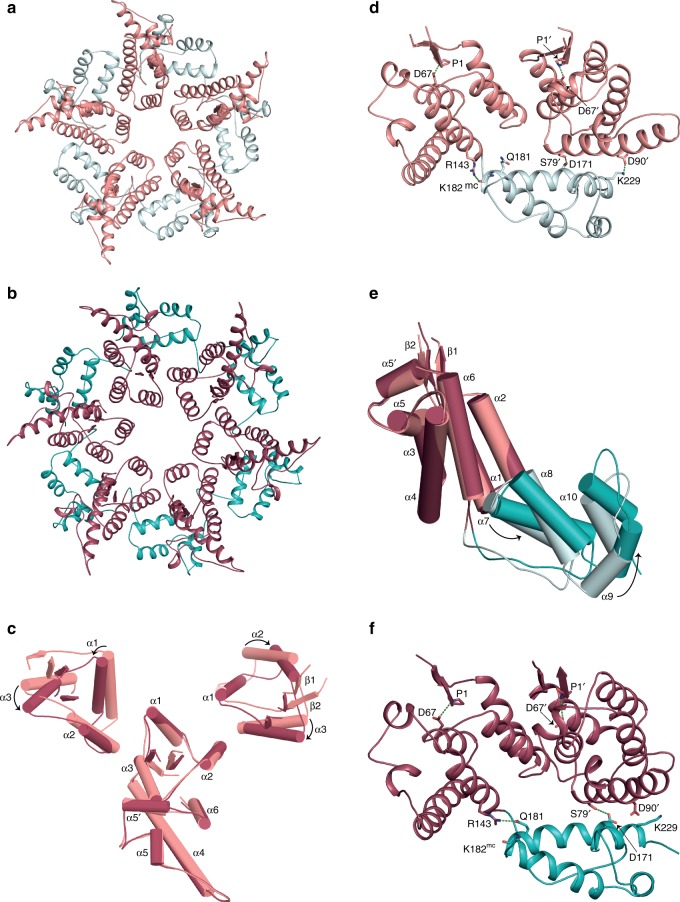
Intra-pentamer and intra-hexamer interactions in HML2 CA^rec^ particles. **a**, **b** Cartoon representations of **a** the T = 1 pentamer and **b** the D6 polar hexamer viewed along the five-fold and six-fold symmetry axes, respectively. The NTDs and CTDs are coloured as in Fig. 2. **c** Alignment of the NTDs from a T = 1 pentamer (pink) and a D6 “polar” hexamer (red). The central NTD is aligned and the arrows indicate the relative displacement of helices α1, α2 and α3 in adjacent monomers at the intra-pentamer or intra-hexamer interface. **d** NTD–CTD interactions in the T = 1 pentamer. **e** NTD domain superposition showing the relative displacement of the CTD in the T = 1 pentamer (pink/cyan) and the D6 “polar” hexamer (red/teal). **f** NTD–CTD interactions in the D6 polar hexamer. In **d**, **f** residues making interactions are shown as sticks, with hydrogen bonds shown as dashes, the prime (′) notation indicates the adjacent NTD and “mc” indicates a main-chain interaction. The conserved N-terminal P1 to α3–D67 interaction is also shown in each NTD for orientation.

In the four shell types, regardless of the surrounding environment, the pentamers adopt an invariant configuration (root-mean-square deviation (RMSD) over 1082 Cα = 0.91 Å) and so a key question is how the CA^rec^ monomer is able to assemble into both pentamers and hexamers. In pentamers and hexamers, the packing of helices α1–α2–α3 around the centre of the ring mediates the inter-protomer NTD interface (Fig. 4a, b), and similar to HIV-1^26^ and MLV^25^, the interface is largely polar, containing solvent facing accessible channels. However, a comparison of the pentamer (Fig. 4a) with the D6 polar hexamer (Fig. 4b), which obeys strict six-fold symmetry, shows that although helix α1–α2–α3 packing is maintained, in hexamers, the CA^rec^ monomers are positioned at a greater radius and are rotated away from the symmetry axis, relative to pentamers. As a result, the relative tilt angle between adjacent α1 helices is reduced and helix α2 comes closer to α3 of the neighbouring monomer allowing the additional monomer to be accommodated into the hexameric ring (Fig. 4c and Supplementary Movie 1).

**Fig. 5:**
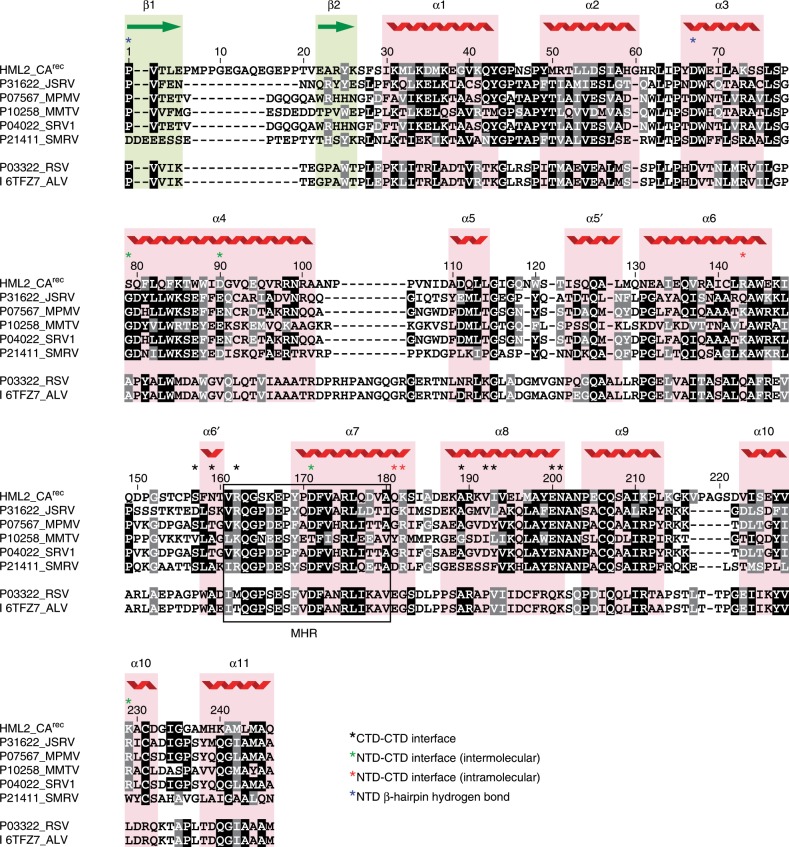
Structure-based sequence alignment of HML2 CA^rec^ with alpha and betaretroviral CAs. The alignment was performed domain-wise using PROMALS3D, numbering refers to the HML2 CA^rec^ sequence and Uniprot accession codes for each aligned sequence are shown on the left. The five betaretroviral: HML2 CA^rec^, JSRV, MPMV, mouse mammary tumour virus (MMTV), Simain type D retrovirus (SRV1) and squirrel monkey retrovirus (SMRV), and two alpharetroviral: RSV and avian leukosis virus (ALV) CAs are grouped separately (upper and lower). The regions corresponding to HML2 CA^rec^ secondary structures are shaded red for α-helices and green for β-strands and also displayed pictorially above the sequences. Residues with ≥50% identity are highlighted in black, and ≥50% similarity in grey. The major homology region (MHR) identified in the CA of all the orthoretroviruses is boxed. Residues in HML2 CA^rec^ that make inter-molecular CTD–CTD, NTD–CTD or intramolecular NTD–CTD contacts and those that form the conserved NTD β-hairpin hydrogen bond are indicated with the coloured asterisks (black, green, red and blue, respectively).

The transformation from pentamer to hexamer also involves remodelling of both the intra-monomer NTD–CTD interface and the CTD interaction with the NTD of the neighbouring monomer in either the pentameric or hexameric ring. In the pentamer, residue R143 located at the C terminus of α6 in the NTD makes a hydrogen bond to the main-chain carbonyl of K182, located at the C terminus of α7 in the CTD. This configuration facilitates formation of a specific hydrogen bonding arrangement between CTD residues D171 on α7 and K229 on α10 with S79′ and D90′ on α4 of the neighbouring monomer NTD (′ denoting a neighbouring monomer) (Fig. 4d) and defines the pentamer conformation. In the D6 hexamer, because of the expanded radius, for the CTD to maintain the interaction with the neighbouring NTD it is rotated by a further 15° and there is an accompanying 2 Å shift and tilt between α6 and α7 (Fig. 4e). This results in an alternative hydrogen-bonding configuration that defines the hexamer conformation where R143 now makes an intra-monomer hydrogen bond with the side chain of Q181 on the penultimate turn of α7, D171 is hydrogen bonded with S79′ but with a shifted geometry and the K229-D90′ interaction is absent (Fig. 4f). Therefore, by selecting one of these hydrogen-bonding configurations, CA^rec^ monomers can adapt to either the pentamer or hexamer conformation. Examination of the sequence conservation of residues making intra-monomer and inter-monomer NTD–CTD interactions (Fig. 5) reveals substantial sequence conservation within the betaretroviruses, extending to a limited extent in the alpharetroviruses. However, conservation of K229-D90′ is betaretrovirus exclusive and appears related to the genera-specific NTD–CTD posture (Fig. 3) that allows the close approach of the CTD to the C-terminal of helix α4 in the neighbouring NTD.

**Fig. 6:**
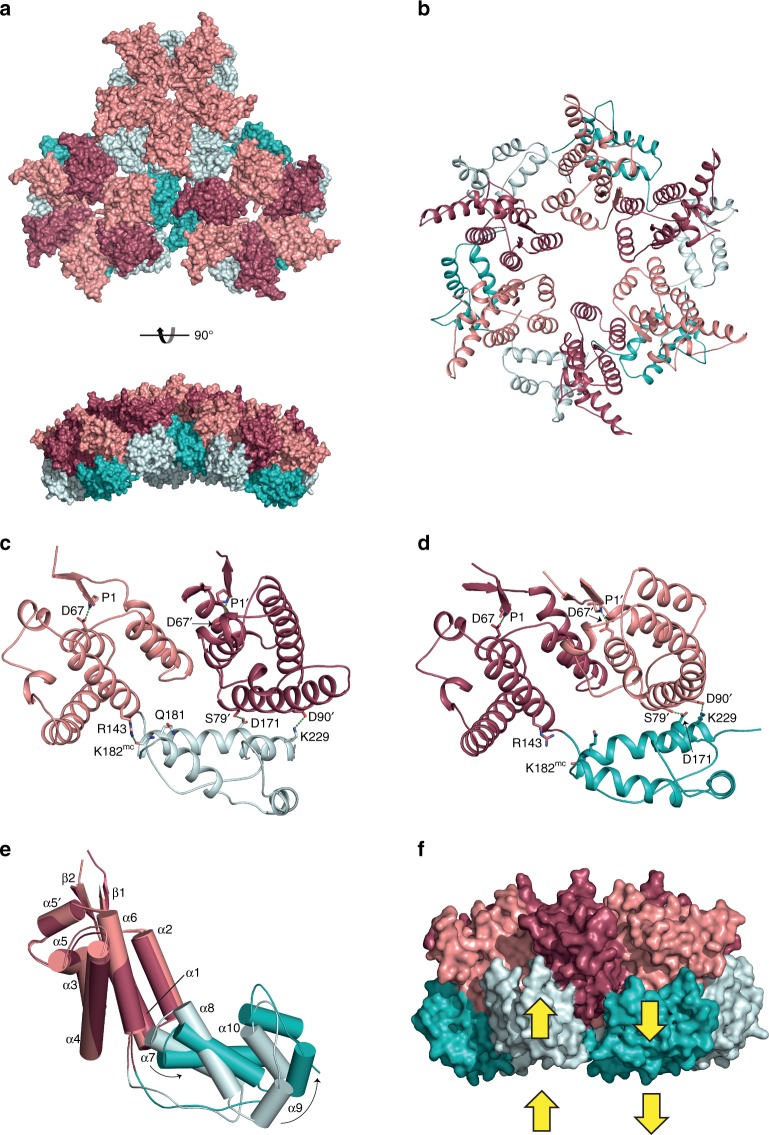
Variation of hexamer–pentamer and hexamer–hexamer interfaces in the *T* = 3 particle. **a** A hexamer–pentamer–hexamer subassembly from the *T* = 3 particle shown in surface representation viewed from the exterior of the shell (upper) and from the side, highlighting the curvature (lower). NTDs and CTDs in the pentamer and those in the hexamer that interface with pentamers are coloured pink and cyan, respectively. NTDs and CTDs in the hexamer that interface with hexamers are coloured red and teal, respectively. **b** Cartoon representation of a hexamer from the *T* = 3 particle viewed down the icosahedral three-fold axis (colouring as in **a**). **c**, **d** NTD–CTD intra-hexamer interactions in the *T* = 3 hexamer. **c** A CA monomer, NTD (pink) and CTD (cyan) that interfaces with a neighbouring pentamer, and the adjacent NTD shown in red. **d** A CA monomer, NTD (red) and CTD (teal) that interfaces with a neighbouring hexamer, and the adjacent NTD is shown in pink. Residues making interactions are shown as sticks with hydrogen bonds shown as dashes, the prime (′) notation indicates the adjacent NTD and “mc” indicates a main-chain interaction. **e** NTD (pink/red) alignment of monomers in *T* = 3 hexamers showing the relative displacement of the CTD when the protomer interacts with either a neighbouring pentamer (cyan) or a neighbouring hexamer (teal). **f** Hexamer surface representation viewed perpendicular to the symmetry axis showing alternate CTDs displaced upwards (cyan, pentamer facing) and downwards (teal, hexamer facing). NTDs are coloured pink or red as in **a**.

In the T = 3 particle, there are no inter-pentamer interactions, instead each pentamer is surrounded by five hexamers. The hexamers contact both pentamers and hexamers through the CTD–CTD interactions with three alternating CA^rec^ protomers linking to T = 3 pentamers and the other three linking to T = 3 hexamers (Fig. 6a). These non-equivalent contacts are accommodated by relative shifts of adjacent CA monomers reducing the hexamer to three-fold symmetry (Fig. 6b), consistent with its location at the icosahedral three-fold axis. Compared to the symmetrical D6 polar hexamer, this creates pairs of monomers with a repacking of their NTD–NTD interfaces and associated shifts in the location of the CTD and its interaction with the NTD of the neighbouring monomer (Fig. 6c–e). When the CTD–CTD interaction is with a pentamer, the intra-hexamer CTD–NTD interaction has the same structural conformation and specific hydrogen bonding arrangement as the T = 1 pentamer conformation (Fig. 6c and Fig. 4d). Notably, all the pentamers in the T = 3 particle also maintain the same intra-pentamer hydrogen bonding arrangement as the T = 1 pentamer, even though each monomer now makes identical interactions with surrounding hexamers rather than pentamers. By contrast, when the CTD–CTD interaction is with a hexamer, the CTD shifts away from its NTD by 3.6 Å, the R143-mediated α6–α7 interaction is lost and the intra-hexamer CTD–NTD interaction has a structural conformation and hydrogen-bonding configuration that is comparable to that of the D6 hexamer (Fig. 6d, e). Thus, the same structural remodelling of inter-monomer interfaces that switches the pentamer to the hexamer conformation is exploited within the T = 3 hexamer to adapt to either the hexamer–pentamer or hexamer–hexamer environment.

This symmetry breaking of the *T* = 3 hexamer is also manifest at the level of whole particle geometry. Measurements of the radial distance of the centroid for *T* = 3 pentamers and hexamers show that pentamers are situated at a higher radius than hexamers, 143.9 and 136.5 Å, respectively. This difference in displacement is accommodated in *T* = 3 hexamers by the alternating shift-up of the CTDs (3.3 Å) to interact with adjacent pentamers and shift-down (3.3 Å) to interact with adjacent hexamers (Fig. 6f). Moreover, adaptation of hexamers to the local environment is also a feature of our other D5 and D6 capsular assemblies, where hexamers interact with each other in the equatorial belt. Again, the pentamer conformation remains invariant and the hexamer six-fold symmetry is broken by NTD domain movements, changes in the NTD–CTD linker conformation and in the intra-hexamer CTD–NTD hydrogen bonding configuration (Supplementary Fig. 10) and in the CTD radial position when interacting with either a neighbouring pentamer or hexamer. These observations regarding the plasticity of the hexamer structure are key when considering the pleotropic assemblies that constitute viral cores and provide an explanation of how CA accommodates the extremes of curvature that are encountered in these structures.

### Building shells through CTD–CTD interactions

**Fig. 7:**
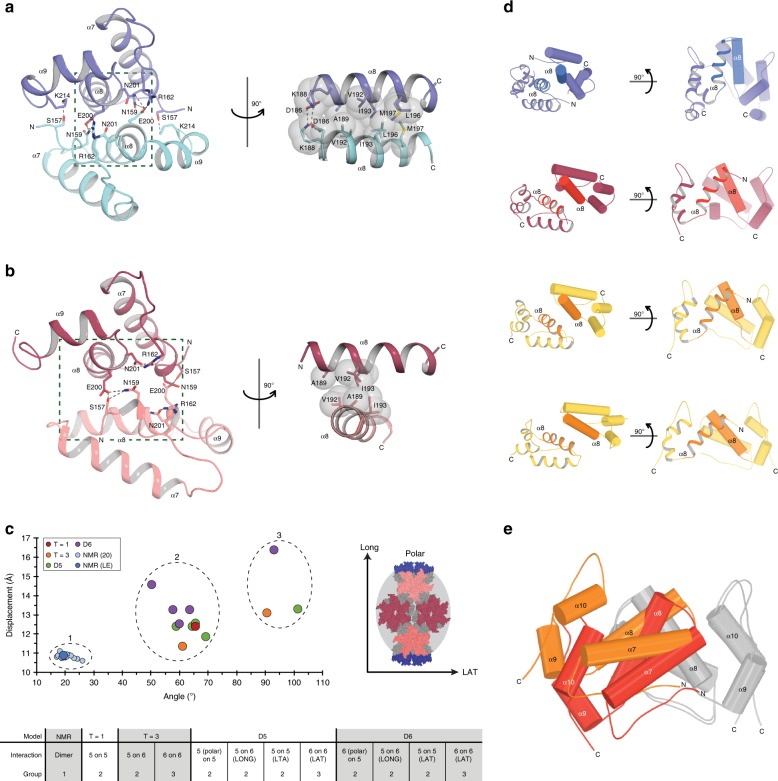
Analysis of HML2 CA^rec^ CTD–CTD interactions. **a**, **b** Structures of the CTD–CTD dimer interface in solution (lowest-energy NMR structure) (**a**) and in the *T* = 1 particle (**b**) are shown in cartoon representation. Residues involved in the interface are shown as sticks with hydrogen bonds shown as dashes. The boxed region is rotated 90° to show the hydrophobic interactions between helix α8 of each monomer, with the van der Waals radii shown as grey space fill. **c** Plot of the cross angles and displacements between α8 helices in all CTD–dimer pairs. The circles are colour-coded with respect to particle type, shown in the key. Groups 1, 2 and 3 are ringed and a schematic describing the nomenclature of POLAR, LONG and LAT is shown alongside. The class of interaction seen in each particle type and the group to which they belong is shown in the key below. **d** Panel of representative CTD dimers from the groups displayed in **c**. Top to bottom: NMR dimer (group 1); *T* = 1 pentamer (group 2); *T* = 3 pentamer on hexamer (group 2); *T* = 3 hexamer on hexamer (group 3). **e** alignment of CTD monomers (grey) showing relative displacement of the interacting CTD monomer in *T* = 1 (red; group 2) and *T* = 3 hexamer on hexamer (orange; group 3).

Given the importance of the CTD inner cage, we also analysed the CTD–CTD interactions that connect pentamers to pentamers, hexamers to pentamers and hexamers to hexamers in all of the Fullerene structures (Fig. 7). To undertake this analysis and as a basis for comparison, we first determined the NMR ensemble structure of the solution dimer and measured HML2 CA^rec^-CTD self-association (*K*_A_ = 3.6 × 10^3^ M^−1^) (Supplementary Fig. 11 and Supplementary Table 4).

The solution structure CTD–CTD interface involves an outer network of polar interactions (residues S157-K214, N159-R162-E200′-N201′ and D186-K188) together with the packing of a core of hydrophobic side chains (residues: A189, V192, I193 and V194, L196, M197) that extend from opposing α8 helices (Fig. 7a). Residues at the CTD interface are largely conserved amongst the betaretroviruses (Fig. 5) and introduction of a I193A/L196A mutant, analogous to the W184A/M185A that disrupts in HIV CTD–CTD interactions^26^, abolishes self-association of HML2 CA^rec^-CTD in solution (Supplementary Fig. 11e).

In this configuration, the α8 helices are oriented with a shallow relative crossing angle of 20° and a helix centroid displacement of 11 Å, allowing extensive hydrophobic contacts along their entire length. By comparison, at the unique type of CTD–CTD interface in the *T* = 1 particle (Fig. 7b) the α8 centroid displacement is maintained at 12 Å, but the crossing angle is significantly enlarged to 65°. This has the effect of twisting the interface relative to the solution state to meet the constraint imposed by icosahedral particle geometry, although remarkably, the hydrophobic interactions of A189, V192 and I193 are preserved, and although much of the polar network is lost, a new compensatory S157–E200–N159 interaction contact is present.

Parameterisation of all the CTD–CTD interactions observed in *T* = 1, *T* = 3, D5 and D6 shells in terms of the crossing angle and α8 centroid displacements provides a way to understand the detailed conformations that CTD–CTD dimers adopt within the particles. These data (Fig. 7c, d) show that the NMR solution ensemble populates one region, group 1, and all family members have α8 crossing angles near to 20° and displacements close to 11 Å. The CTD–CTD dimers in the particles fall into two other classes: group 2 that contains all CTD–CTD interactions at pentamer–pentamer and pentamer–hexamer interfaces and have 65° crossing angles and displacements between 11 and 15 Å, and group 3 that contains all the CTD–CTD interactions at hexamer–hexamer interfaces and have crossing angles near to 95° and displacements between 13 and 16 Å. T = 3 particles have two CTD–CTD configurations, a group 2 set from hexamer–pentamer interfaces with a similar configuration to that observed at *T* = 1 pentamer interfaces and a group 3 set with the larger crossing angle found at hexamer–hexamer interfaces (Fig. 7d, e). In the D5 and D6 particles, further configurations are possible depending on whether pentamers and hexamers are located at poles or are equatorial and interact either longitudinally or laterally (Fig. 7c). Nevertheless, all hexamer–pentamer interfaces still fall into group 2 and all hexamer–hexamer interfaces still fall into group 3, albeit in D6, the α8 displacement for the equatorial hexamer–hexamer interactions are larger than in D5, due to the lower curvature of the equatorial region.

Previously, a coarse-grained theoretical model for a larger HIV-1 fullerene cone-shaped capsid^34^ was constructed with a continuum of helix α8 crossing angles covering the range of groups 2 and 3 observed in HML2 CA^rec^. Our data now demonstrate how discretisation of crossing angles between CTDs facilitates adaptation of CA to the different environments, particularly at the points of curvature that pentamers introduce into the structures.

## Discussion

Our studies of HML2 CA^rec^ show that an ERV retains the ability to form capsid assemblies using the same architecture as exogenous viruses. The full diversity of interactions we observe in our structures reveals (1) plasticity of packing at the NTD–NTD interfaces, (2) alternative geometry and bonding configurations at the NTD–CTD interfaces and (3) the quantisation of the CTD–CTD association parameters. Plasticity at the NTD–NTD interface has been observed previously in crystal structures of bovine leukaemia virus CA^28^, where hexamers are distorted from perfect 6-fold geometry to allow the CTD–CTD interfaces to maintain a hexagonal lattice that would otherwise be dislocated by crystal packing forces. Further, flexibility has been observed in intact HIV-1 virion cores^29^ where small rotations at the interdomain linker and CTD–CTD interface are distributed over the whole core to accommodate differences in curvature of the Fullerene cone. Now our high-resolution structures enable us to classify and quantify these molecular interactions and provide a rule book for HML2 CA^rec^ pentamer and hexamer construction. Moreover, they define how switching of the intra-hexamer NTD–CTD configurations facilitates symmetry breaking of the hexamers to adapt to distinct local environments by positioning the CTD for interaction with the CTD of the adjacent pentamer or hexamer. This provides the basis for building structures with a wide range of shapes and sizes consistent with Fullerene geometry and more pleomorphic morphologies found in exogenous betaretroviruses, the highly similar alpharetroviruses and the more distant lentiviruses.

## Methods

### Details of constructs

A DNA sequence coding for the consensus HML2 CA^rec^ protein was synthesised codon optimised for *E. coli* expression (Geneart) (Supplementary Fig. 1). The sequence coding for the full-length (HML2 CA^rec^; P1-Q246), amino terminal (P1-P151; CA^rec^-NTD) and C-terminal domain (P156-Q246; CA^rec^-CTD) were amplified by PCR and the products inserted into a pET22b expression vector (Novagen) between the *Nde*I and *Xho*I restriction sites in order to produce C-terminal hexa-histidine fusions. The I193A/L196A double mutation was introduced into CA^rec^-CTD using the Quikchange II Site-directed Mutagenesis Kit (Agilent) according to the manufacturer’s instructions. The correct sequence of expression constructs was verified by automated DNA sequencing (Beckman Coulter Genomics), primer sequences used for PCR cloning and mutagenesis are presented in Supplementary Table 5. Proteins were expressed in the *E. coli* strain BL21 (DE3) by the addition of 0.1 mM isopropyl β-d-1-thiogalactopyranoside (IPTG) to log-phase cultures followed by continued growth at 20 °C overnight. The bacteria were harvested and resuspended in 10 mL of lysis buffer per gram of cells (50 mM Tris, pH 8, 250 mM NaCl, 10 mM imidazole, 5 mM MgCl_2_, 0.5 mM TCEP (Tris(2-carboxyethyl)phosphine), 0.2% v/v Triton X-100). The cells were lysed by sonication and the clarified supernatant was injected onto a 5 mL HisTrap Column (GE Healthcare). Bound sample was washed with wash buffer (50 mM Tris, pH 8, 750 mM NaCl, 20 mM imidazole, 5 mM MgCl_2_, 0.5 mM TCEP, 0.2% v/v Triton X-100, 4 mM ATP) and His A buffer (20 mM Tris, pH 8, 250 mM NaCl, 10 mM imidazole, 0.5 mM TCEP) and eluted with His B buffer (20 mM Tris, pH 8, 250 mM NaCl, 500 mM imidazole, 0.5 mM TCEP). For HML2 CA^rec^-CTD, the eluent was concentrated to ~3 mL and the protein further purified by SEC on a Superdex 75(26/60) column equilibrated in SEC buffer (20 mM Tris pH 8, 100 mM NaCl, 0.5 mM TCEP). For HML2 and CA^rec^ HML2 CA^rec^-NTD the eluents from HisTrap column were diluted 25-fold in IEX A buffer (20 mM Tris pH 8, 0.5 mM TCEP) and applied a 6 mL Resource Q ion exchange column. Proteins were eluted using a 40 column-volume gradient into IEX B buffer (20 mM Tris pH 8, 1 M NaCl, 0.5 mM TCEP). Fractions containing HML2 CA^rec^ or HML2 CA^rec^-NTD were concentrated to ~3 mL and further purified by SEC on a Superdex 200(26/60) column equilibrated in SEC buffer. All purified proteins were concentrated to 15–30 mg mL^−1^, flash frozen in liquid nitrogen and stored at −80 °C. Selenium was incorporated into the N-terminal domain CA^rec^-NTD construct by replacement of methionine with seleno-methionine in defined culture medium and by inhibition of methionine biosynthesis just prior to IPTG induction^38^. For CA^rec^-CTD NMR experiments, ^15^N and ^13^C-^15^N uniformly labelled protein was expressed in M9 minimal media with ^15^NH_4_Cl or ^15^NH_4_Cl and ^13^C_6_-glucose, as required, as sole nitrogen or nitrogen and carbon sources. To obtain triple-labelled, ^2^H-^13^C-^15^N samples, the M9 media containing ^15^NH_4_Cl and ^13^C_6_-glucose was prepared in ^2^H_2_O instead of H_2_O. Isotopically labelled and selenium incorporated samples were purified in the same way as unlabelled protein. Verification of N-terminal methionine processing, correct molecular mass, degree of selenium and isotopic label incorporation was obtained by electrospray ionisation mass spectrometry.

### SEC-coupled multi-angle laser light scattering

SEC-MALLS was used to estimate the molar mass of HML2 CA^rec^ assemblies. Samples (100 µL) ranging from 2 to 11 mg mL^−1^ of HML2 CA^rec^ were applied to a Superose 6 10/300 GL column equilibrated in 20 mM Tris-HCl, 1 M NaCl, 0.5 mM TCEP, pH 8.0, at a flow rate of 0.3 mL min^−1^ at 25 °C. The scattered light intensity and the protein concentration of the column eluate were recorded using a DAWN-HELEOS laser photometer and OPTILAB-rEX differential refractometer respectively. The weight-averaged molecular mass of materials contained in chromatographic peaks were determined from the combined data from both detectors using the ASTRA software version 6.0.3 (Wyatt Technology Corp., Santa Barbara, CA, USA).

### Cryo-EM sample preparation and data collection

HML2 CA^rec^ (16 mg mL^−1^) was adjusted with high salt buffer (20 mM Tris-HCl, 5 M NaCl, 0.5 mM TCEP, pH 8.0) to final salt and protein concentrations of 1.4 M and 10 mg mL^−1^, respectively. Samples were incubated at 4 °C for 1 h prior to plunge freezing. Quantifoil R2/2 200 mesh copper grids were prepared by glow discharge at 25 mA for 1 min in air (EMITECH). All grids were frozen using a Vitrobot mark III at 4 °C and 100% relative humidity. Two-microlitre sample was added to carbon side of grid and incubated for 30 s in the Vitrobot chamber before blotting for 0.5 s. Immediately after blotting, 2 µL of low salt buffer (20 mM Tris, pH 8, 100 mM NaCl) was applied, followed by a 5 s blot and plunge freezing into liquid ethane. Data from frozen-hydrated samples were collected on a Titan Krios (Thermo Fisher) in nanoprobe mode on a Falcon III detector operating in counting mode. Movies were collected at a magnification corresponding to a calibrated pixel size of 1.09 Å. Movies were recorded with an exposure time of 59.4 s, corresponding to 30 frames and a total dose of 30 e^−^ Å^−2^.

### Image processing

All movies were motion corrected on the fly using Scipion v1.1^39^ with exposure weighting in MotionCor2^40^. Initial parameters for the contrast transfer function correction were estimated from non-exposure-weighted sums in CTFFIND4.1^41^. Micrographs with a quality of fit better than 7 Å resolution were selected for further analysis. A total of 1,498,437 particles from 10,935 dose-weighted micrographs were identified by template-based particle picking using RELION 2.1^42^. Two cycles of reference-free 2D classification yielded 1,218,119 particles for further analysis. The particle classes were further subdivided based on particle diameter guided by known sizes for spherical capsular particles from prior negative stain studies: 715,082 (T = 1), 211,787 (D5 and D6) and 398 (T = 3).

For the reconstruction of the T = 1 particle, an ab initio model without symmetry was generated from a subset of particles (20,000) using cryoSPARC v.2^43^. Two cycles of 3D classification were carried out to identify best particles for subsequent refinement. 3D auto-refinement in RELION 2.1 imposing I2 symmetry was performed on 121,357 particles belonging to the most populated, highest-resolution 3D classes, followed by 3D classification without alignment and auto-refinement of the highest resolution class. Particles and the map corresponding to the best class were then subject to manual refinement of all parameters using cisTEM^44^ with an initial high-resolution limit for refinement set to 10 Å and final limit of 5 Å. The final reconstruction, performed on 64,731 particles extracted into a 576 × 576 pixel box, has a resolution of 2.75 Å (0.143 Fourier shell correlation (FSC) threshold criterion^45^).

For the reconstruction of the D5 and D6 capsular particles, an ab initio model was generated without symmetry in cryoSPARC using a subset of particles. One cycle of 3D classification yielded two distinct species of capsular structures. Particles belonging to each species were separated into two stacks of 153,442 (D5) and 88,345 (D6) particles, respectively. Ab initio models were generated using cryoSPARC for each stack separately and further cycles of 3D classification in RELION, were performed both without symmetry as well as imposing D5 symmetry for the 153,442-particle stack and D6 symmetry for the 88,345-particle stack. For the D5 particle, a final stack of 93,221 particles was first refined in cryoSPARC using a homogeneous refinement procedure and then manually refined in cisTEM with an initial high-resolution limit set at 10 Å and final limit of 5 Å. Final refinements and reconstructions were performed on particles extracted into a 648 × 648 pixel box size. This produced a final map with resolution of 3.18 Å (0.143 FSC threshold criterion). For the D6 particle, a final stack of 16,723 particles was refined in an identical manner using cisTEM to produce a final map at 3.77 Å resolution (0.143 FSC threshold criterion).

For the remaining 398 particles from the original 2D classification showing T = 3 icosahedral symmetry, ab initio model generation in cryoSPARC and homogeneous refinement yielded a map of 4.66 Å resolution (0.143 FSC threshold criterion) from 359 particles. Manual refinement of this structure and corresponding particles in cisTEM extracted into a 648 × 648 pixel box size using a high-resolution limit for refinement set to 10 Å. In the last cycle of refinement, a spherical mask with a radius of 100 Å to remove the inner density further improved the map to 4.34 Å (0.143 FSC threshold criterion). Refinement of the inner layer was performed on the same stack of 359 particles, using a T = 1 map lowpass filtered to 40 Å with a mask around the T = 1 map to exclude the outer T = 3 density and refined imposing I2 symmetry. This produced a final map of a T = 1 particle to 5.24 Å (0.143 FSC threshold criterion).

### Model building

Maps from the T = 1 and D5 particles were segmented using the Seggar tool, implemented in UCSF Chimera^46^ to extract density for a pentamer (T = 1) and hexamer (D5), respectively. The extracted map segments were then used for model building. Initially, the CA^rec^-NTD and CA^rec^-CTD structures were docked into density using rigid body refinement in chimera. The fits were then further optimised using the JiggleFit tools and missing sections of the model built into density in COOT^47^ with iterative rounds of real-space refinement in PHENIX^48^. The refined monomer structure from the pentamer and each unique interface of the hexamer were then used to construct appropriate asymmetric units for each capsid assembly. Whole particle structures were then further refined with NCS using PHENIX real-space refine to produce final refined models for each map. All models comprise residues 1–6 and 23–231, but with varying completeness in the region 149 to 156. The local resolution of maps was determined using ResMap^49^ and models were validated throughout refinement using MolProbity^50^ and quality of fit assessed using map vs. model FSC in PHENIX. Final structures were symmetry expanded in chimera to produce overall assemblies. Details of data collection and model refinement are presented in Supplementary Table 1.

### Protein crystallisation and structure determination

HML2 CA^rec^-NTD was crystallised using hanging drop vapour diffusion. Typically, a 40 mg mL^−1^ solution of HML2 CA^rec^-NTD in 150 mM NaCl, 20 mM Tris-HCl and 0.5 mM TCEP, pH 8.0, was mixed with an equal volume of crystallisation solution containing 0.2 M sodium acetate trihydrate, 0.1 M Tris hydrochloride and 15% (w/v) PEG 4000 and suspended over a reservoir of the crystallisation solution. Drops were incubated at 18 °C, crystals appeared within 2–3 days and were transferred into fresh crystallisation solution supplemented with 20% (v/v) glycerol and flash frozen in liquid nitrogen. The crystals belonged to either the space group *C222*_*1*_ with three copies of HML2 CA^rec^-NTD in the asymmetric unit (AU) or space group *C2* with two copies of HML2 CA^rec^-NTD in the AU. Seleno-methionine-derived protein was crystallised under the same conditions.

The structure of HML2 CA^rec^-NTD was solved by a combination of single wavelength anomalous diffraction (SAD) and molecular replacement. Initially a 3.2 Å dataset was recorded from a C222_1_ orthorhombic crystal of the seleno-methionine substituted protein at a wavelength of 0.9791 Å and 100 K on beamline I03 at the Diamond Light Source (Didcot, UK). Data were processed using the HKL program package^51^ and 21 selenium atoms were located using SAD methods implemented in SHELX^52^. Best phases were calculated using only 9 selenium atoms together with non-crystallographic averaging and density modification in PHENIX, resulting in a Figure of Merit of 0.67 and a map of sufficient quality to unambiguously build a near complete model of a single protomer using Arp/Warp^53^. A further high-resolution 1.8 Å dataset was collected on a C2 monoclinic crystal at 100 K using Cu Kα radiation from an in-house X-ray source (Rigaku Micromax-007HF with a Raxis-IV detector). The monomer from the C222_1_ crystal was then employed as a molecular replacement search model with this C2 dataset. Molecular replacement was carried out using Phaser^54^ and located the two copies of HML2 CA^rec^-NTD in the AU. The model was completed by iterative rounds of reciprocal space refinement in PHENIX and model building in COOT. TLS groups were determined using TLSMD^55^ and were included in the final round of refinement.

The final model comprises residues 1–6 and 23–151 (chain A) and residues 1–5 and 21–149 (chain B) and was refined to a R_work_/R_free_ of 15.7/19.5%. The model quality was assessed using Molprobity and has good geometry with 98.8% of residues in the preferred region of the Ramachandran plot, only 1.2% in the additionally allowed region and no outliers. Details of crystal parameters phasing and data refinement statistics are presented in Supplementary Table 2.

### NMR spectroscopy and structure determination

NMR experiments were recorded at 298 K on Bruker Avance 600-, 700-, 800- and 950-MHz spectrometers. ^1^H-^15^N, ^1^H-^13^C-^15^N and ^2^H-^13^C-^15^N-labelled CA^rec^-CtD samples were prepared in buffer containing 20 mM Tris-HCl, 50 mM NaCl and 0.5 mM TCEP, pH 7.0. Protein concentration for the NMR experiments was ~2.3 mM. ^1^H, ^13^C and ^15^N resonance assignments for protein backbone were obtained from three-dimensional HNCA, HN(CO)CA, HNCO, HN(CA)CO and HNCACB, recorded on ^2^H-^13^C-^15^N-labelled samples, and from HN(CO)CACB, CBCA(CO)NH and HNCANNH experiments recorded on ^13^C-^15^N-labelled samples. Side chain resonances were assigned using ^13^C-^15^N-labelled samples and, for aliphatic proteins, 3D HBHA(CO)NH, CC(CO)NH, H(CCO)NH, (H)CCH-TOCSY and CCH-TOCSY spectra. Aromatic protons were instead assigned from the analysis of ^1^H-^13^C HSQC (heteronuclear single quantum coherence) and 3D ^13^C-edited NOESY-HSQC (NOE spectroscopy-HSQC) experiments tuned to aromatic carbons as well as 2D (HB)CB(CGCD)HD and 2D (HB)CB(CGCDCE)HE experiments. Inter-proton distance restraints for structural calculations were obtained from 3D ^13^C-edited NOESY-HSQC and ^15^N-edited NOESY-HSQC spectra recorded using a 100 ms mixing time from a fully protonated ^13^C-^15^N-labelled sample. The dimer interface of CA^rec^-CTD was identified by inter-molecular distance restraints using F1-^13^C/^15^N-filtered, F3-^13^C-edited NOESY-HSQC spectra recorded with a 150 ms mixing time. The 3D-filtered spectra were obtained using an asymmetrically labelled dimer of CA^rec^-CTD prepared by mixing equimolar unlabelled protein with uniformly ^13^C/^15^N-labelled protein (2 mM total protein concentration). Hydrogen bonds were identified on the basis of preliminary structure calculations and confirmed from analysis of CLEANEX-PM^56^ and HNCO-long range^57^ experiments. All spectral data were processed with NMRPipe^58^ and analysed with CARA^59^.

Backbone ^15^N relaxation measurements of T_1_ spin–lattice relaxation time, T_2_ spin–spin relaxation time and the steady-state heteronuclear ^1^H-^15^N NOE of HML2 CA^rec^-CTD were recorded at 298 and 278 K on a 700 MHz spectrometer using 0.3 mM ^15^N-labelled samples. The time delays for T_1_ experiments were 10, 50, 100, 200, 400, 500, 750, 1000 and 1400 ms, and for T_2_ experiments were 8, 16, 32, 48, 64, 80, 96, 112, 128 and 160 ms. T_1_ and T_2_ relaxation data were obtained by fitting individual peak intensities using nonlinear spectral lineshape modelling to a single exponential using routines within NMRPipe. ^1^H-^15^N NOE values were calculated from peak intensity ratios obtained from spectra with and without ^1^H saturation prior to the ^15^N excitation pulse. The average errors were estimated at 3% for the T_1_ and T_2_ measurements and at 5% on the steady-state heteronuclear ^1^H-^15^N NOE values.

The solution structure of HML2 CA^rec^-CTD was calculated using the program ARIA (Ambigious Restraints for Iterative Assignment v 2.3)^60^. Briefly, nine iterations of a simulated annealing protocol were performed where progressive NOE cross-peak assignment and conversion in the structure(s) calculation process were achieved based on NOE distance restraints and, for helices α7–α10, hydrogen bond and dihedral angle restraints as predicted by the program TALOS+^61^. The inter-molecular distance restraints defining the HML2 CA^rec^-CTD homodimer interface were derived from inter-proton NOE correlations observed in the filtered NOESY experiments, while the corresponding NOEs were removed from the 3D ^13^C-NOESY-HSQC constraint list to avoid duplications. A set of 100 structures were calculated and the 20 lowest-energy structures of the set were taken to represent the converged ensemble and refined in an explicit water shell. The superimposition of the 20 water-refined structures is shown in Supplementary Fig. 11b. The quality of the calculated structure ensemble was assessed and validated with the Protein Structure Validation Suite-PSVS^62^ and Procheck-NMR^63^. For the final 20 lowest-energy NMR structures, no distance or torsional angle restraint was violated by more than 0.5 Å or 5°, respectively. The Ramachandran plot for the family of structures showed 88% of residues are in the most favoured region, 11% are in the additionally allowed region and only 1% are outliers. Details of the structure determination are summarised in Supplementary Table 3.

### 3D structural alignments and interface analysis

The European Bioinformatics Institute (EBI) protein structure comparison service (PDBeFold) (http://www.ebi.ac.uk/msd-srv/ssm/) was used to perform searches with HML2 CA^rec^-NTD and HML2 CA^rec^-CTD for structural homologues in the PDB. Orthoretroviral CA-NTDs and -CTDs comprised > 95 % of the top 50 hits. The fit qualities based on RMSD of Cα positions were ranked using the Z-score. Molecular interfaces were analysed using the EBI protein structure interface analysis service PDBePISA (http://www.ebi.ac.uk/msd-srv/prot_int/). Analysis of CTD–CTD crossing angle and displacements were performed as follows. Centroids of α8 helices were obtained by calculating the average *x*, *y*, *z* positions of all backbone atoms (N, CA, C) in the helix. Centroids for the “first half” of the helix and the “second half” of the helix were calculated and used as the start and end points of the vector. Angles between the vectors were obtained by calculating the dot product of the two vectors and dividing by the product of the vector magnitudes.

### Structure-based sequence alignment

Structure-based sequence alignment of alpha and betaretroviral CA-NTDs and CA-CTDs was performed using the PROMALS3D^64^ server. Where necessary, alignments were adjusted manually based on the position of secondary structures observed in seed structures used for the individual CA-NTD and CA-CTD alignments.

### Analytical ultracentrifugation

Sedimentation velocity experiments were performed in a Beckman Optima Xl-I analytical ultracentrifuge using conventional aluminium double sector centrepieces and sapphire windows. Solvent density and the protein partial specific volumes were determined from tabulated values^65^. Prior to centrifugation, samples were prepared by exhaustive dialysis against the buffer blank solution, 20 mM Tris-HCl, 2 M NaCl and 0.5 mM TCEP, pH 8.0. Centrifugation was performed at 20,000 r.p.m. (29,120 × *g*) and 293 K in an An-50-Ti rotor. Interference data were acquired at time intervals of 180 s at a sample concentration of 1.5 mg mL^−1^. Data recorded from moving boundaries were analysed using the size distribution function C(S) in the program SEDFIT^66–68^.

Sedimentation equilibrium experiments were performed in a Beckman Optima XL-I analytical ultracentrifuge using aluminium double sector centrepieces in an An-50 Ti rotor. Prior to centrifugation, samples were dialysed exhaustively against the buffer blank 20 mM Tris-HCl, pH 8, 150 mM NaCl and 0.5 mM TCEP. After centrifugation for 30 h at 22,000 r.p.m. (35,235 × *g*), interference data was collected at 2 hourly intervals until no further change in the profiles was observed. The rotor speed was then increased to 26,000 rpm (49,213 × *g*) and then 30,000 r.p.m. (65,520 × *g*) and the procedure repeated each time. Data were collected on samples of HML2 CA^rec^-CTD and CA^rec^-CTD (I193A/L196A) at varying protein concentration (50–200 µM) and at three speeds. The program SEDPHAT^69,70^ was used to determine weight-averaged molecular masses by nonlinear fitting of individual multi-speed equilibrium profiles to a single-species ideal solution model. Inspection of these data revealed that the HML2 CA^rec^-CTD molecular mass showed significant concentration dependency and so global fitting to a monomer–dimer equilibrium model incorporating the data from the three speeds and three sample concentrations was applied to extract the dimerisation association constant (*K*_A_). Details of protein hydrodynamic parameters and sedimentation equilibrium data are presented in Supplementary Table 4.

To compare the self-association properties of HML2 CA^rec^-CTD with the dimer interface mutant HML2 CA^rec^-CTD (I193A/L196A), MSTAR analysis was performed on the equilibrium distributions recorded at 30,000 r.p.m. (65,520 × *g*) on 200 µM samples using the program SEDFIT-MSTAR^71^. The point average molecular weight (*M*_w_*) showed the expected increase with increasing radius for CA^rec^-CTD and yielded a weight-averaged molecular mass at the cell bottom (*M*_w,b_) intermediate between monomer and dimer. For CA^rec^-CTD (I193A/L196A), no radial dependency of *M*_w_* was observed and the *M*_w,b_ obtained equated to monomer molecular mass.

### Reporting summary

Further information on research design is available in the Nature Research Reporting Summary linked to this article.

## Supplementary information

Supplementary Information

Description of Additional Supplementary Files

Supplementary Movie 1

Reporting Summary

## Data Availability

The structural data that support the findings of this study have been deposited in the Protein Data Bank, BioMagResBank and EM Data Bank. The coordinates for HML2 CA^rec^-NTD and HML2 CA^rec^-CTD have the PDB accession numbers 6SA9 and 6SAI, respectively. Chemical shift assignments for HML2 CA^rec^-CTD in the BioMagResBank have the accession number 34419. The coordinates for HML2 CA^rec^ *T* = 1, D5, D6 and *T* = 3 assemblies have PDB accession numbers 6SSJ, 6SSK, 6SSL and 6SSM and the EM maps have EMDB entry numbers EMD-10295 [https://www.ebi.ac.uk/pdbe/entry/emdb/EMD-10295], EMD-10296 [https://www.ebi.ac.uk/pdbe/entry/emdb/EMD-10296], EMD-10297 [https://www.ebi.ac.uk/pdbe/entry/emdb/EMD-10297] and EMD-10298 [https://www.ebi.ac.uk/pdbe/entry/emdb/EMD-10298], respectively. Other data are available from the corresponding authors upon reasonable request.
