# Supplementary material for: Structural basis for Fullerene geometry in a human endogenous retrovirus capsid
Source: Nat Commun. 2019 Dec 20;10:5822. doi: 10.1038/s41467-019-13786-y (PMC6925226; doi:10.1038/s41467-019-13786-y)
Supplement: Supplementary file 1 — Supplementary Information [file 41467_2019_13786_MOESM1_ESM.pdf]

Supplementary Information

Structural Basis for Fullerene Geometry in a Human Endogenous Retrovirus Capsid

Oliver Acton *et al*,

Supplementary Table 1. HML2-CA^{rec} cEM map and model refinement statistics

	T=1	D5	D6	T=3
**Data collection**				
Magnification	75,000x	75,000x	75,000x	75,000x
Voltage (kV)	300	300	300	300
Total exposure (e^{-} Å^{-2})	30	30	30	30
Defocus range (μm)	-1.0 to -3.5	-1.0 to -3.5	-1.0 to -3.5	-1.0 to -3.5
Pixel size (Å)	1.09	1.09	1.09	1.09
Initial particle stack	715,082	153,442	88,345	398
Final particle stack	64,731	93,221	16,723	359
Map resolution (0.143 FSC threshold) (Å)	2.75	3.18	3.77	4.34
**Model refinement**				
Model resolution (0.5 FSC threshold) (Å)	3.0	3.4	4.1	4.9
Map B-factor	-90	-90	-90	-90
Model composition				
Nonhydrogen atoms	103,860	156,280	185,208	307,680
Protein residues	13,320	19,910	23,580	39,240
Ligand	0	0	0	0
B-factors (Å^2)				
Protein	30.75	79.07	154.47	206.56
R.M.S. deviations				
Bond lengths (Å)	0.016	0.008	0.008	0.006
Bond angles (°)	1.381	0.879	1.240	1.236
Validation				
Molprobity Score	1.21	1.30	1.73	1.81
Clash score	2.31	2.57	6.11	9.05
Poor rotomers (%)	0.54	0.18	0.42	0.72
Ramachandran				
Favoured (%)	96.79	96.15	95.16	95.33
Allowed (%)	3.21	3.54	4.48	4.36
Disallowed (%)	0.00	0.31	0.36	0.31
CaBLAM outliers (%)	1.87	2.41	3.16	2.06

Supplementary Table 2. HML2 CA^{rec}-NTD X-ray data collection phasing and refinement statistics

	Se (SAD)	
**Data collection**		
Space group	$C222_1$	C2
Cell dimensions		
a, b, c (Å),	46.0, 231.7, 120.5	42.4, 98.9, 76.5
α, β, γ (°)	90.0, 90.0, 90.0	90.0, 94.4, 90.0
Wavelength (Å)	0.979	1.5418
Unique reflections	20476 (anom)	26836
Resolution range (Å)	25-3.2 (3.31-3.2)*	35-1.8 (1.86-1.8)
R_{sym} (%)	8.4 (37.5)	5.0 (24.5)
I / σI	26.4 (3.7)	35.9 (8.7)
Completeness (%)	99.5 (96.6)	92.2 (71.4)
Redundancy	8.8 (6.9)	7.1 (6.5)
**Phasing**		
No. sites	9	
FOM (solve)	0.44	
FOM (resolve)	0.67	
MapCC	0.56	
**Refinement**		
Resolution (Å)		25-1.8 (1.86-1.8)
R_{work}/R_{free} (%)		15.7/19.5 (16.8/22.3)
No. residues/atoms		
Protein		2280
Water		310
Glycerol		6
B-factors (Å^2)		
Wilson		21.4
Protein		25.0
Water		39.4
Overall		26.7
R.M.S. deviations		
Bond lengths (Å)		0.006
Bond angles (°)		0.977

*Values in parentheses are for highest-resolution shell.

Supplementary Table 3. HML2 CA^{rec}-CTD NMR data and refinement

	HML2 CA^{rec}-CTD
**NMR distance and dihedral constraints**	
NOE Distance constraints	
Total NOE	3616
Unambiguous	3108
Intermolecular	59x2
Hydrogen bonds	27x2
Total dihedral angle restraints	
φ	76x2
Ψ	76x2
**Structure statistics**	
Violations (mean and s.d.)	
Distance constraints (>0.5Å)	0
Max. distance constraint violation (Å)	0.3
Deviations from idealised geometry	
Bond lengths (Å)	0.009±0.001
Bond angles (°)	3.4±0.1
Improper (°)	0.41±0.06
Average pairwise r.m.s. deviation (Å)	
Backbone (all residues)	0.47±0.17
Heavy (all residues)	0.88±0.19

Supplementary Table 4. HML2 CA^{rec}-CTD Sedimentation equilibrium

Protein Parameter				
v ($ml.g^{-1}$)	0.732			
ρ ($g.ml^{-1}$)	1.005			
[a]M_r	10,903			
[b]ε_{280} ($M^{-1}.cm^{-1}$)	3900 (3250)[c]			
[d]j_{inc} ($M^{-1}.cm^{-1}$)	35,980 (29,983)			
Sedimentation Data				
C (μM)	50	100	200	[e]100-200
[f]M_w (kDa)	15.4	16.2	17.8	15.4-17.8
[g]Log_{10} K_A	3.75	3.79	3.35	3.56
[h]K_A (M^{-1})	$5.62x10^3$	$6.17x10^3$	$2.24x10^3$	$3.63x10^3$
[i]K_D (M)	$1.78x10^{-4}$	$1.62x10^{-4}$	$4.47x10^{-4}$	$2.75x10^{-4}$
[j]r.m.s.d.	0.0057	0.0086	0.0043	0.007-0.0081
[k]χ^2	1.32 (4356)	2.14 (4086)	1.68 (4041	2.32 (12483)

[a]molar mass calculated from the protein sequence
[b]molar absorbance extinction coefficient
[c]values in parenthesis are for a 12 mm AUC cell.
[d]molar fringe increment.
[e]global sedimentation equilibrium fit data, combining all three concentrations at three speeds.
[f]weight averaged molecular weight derived from global fits using a single species model.
[g]Log_{10} equilibrium association constant from global fitting to a monomer-dimer self-association model.
[h]equilibrium association constant from global fitting to a monomer-dimer self-association model.
[i]equilibrium dissociation constant from global fitting to a monomer-dimer self-association model.
[j]rms deviation of the data obtained from fitting each multi-speed sample to a monomer-dimer self-association model.
[k]reduced chi-squared for the global fitting, values in parenthesis are the number of data points fitted.

Supplementary Table 5. Primers for HML2 CA cloning and mutagenesis

Construct		Primers (5' -3')*
CA^{rec}	FWD	GACTCATATGCCGGTGACCCTGGAAC
	REV	GACTCTCGAGCTGCGCCATCAGCATG
CA^{rec}-NTD	FWD	GACTCATATGCCGGTGACCCTGGAAC
	REV	GACTCTCGAGCGGGTCCTGAATTTTTTCCC
CA^{rec}-CTD	FWD	GACTCATATGCCGAGCTTTAACACCGTG
	REV	GACTCTCGAGCTGCGCCATCAGCATG
CA^{rec}-CTD (I193A/L196A)[#]	FWD	GAAAAAGCGCGTAAAGTGGCTGTGGAAGCGATGGCGTATGAAAACGCG
	REV	CGCGTTTTCATACGCCATCGCTTCCACAGCCACTTTACGCGCTTTTTC

*Restriction sites used for cloning are underlined

[#]Mutagenized codons are highlighted and underlined

HML2-CA^{rec} DNA coding sequence

ATGCCGGTGACCCTGGAACCGATGCCGCCGGGTGAAGGTGCGCAGGAAGGCGAACCGCCGAC
CGTGGAAGCGCGTTATAAATCGTTCAGCATCAAAATGCTGAAAGATATGAAAGAAGGCGTGA
AACAGTACGGCCCGAACAGCCCGTATATGCGTACCCTGCTGGATAGCATTGCGCATGGCCAT
CGTCTGATTCCGTATGATTGGGAAATTCTGGCCAAAAGCAGCCTGAGCCCGAGCCAGTTTCT
GCAGTTTAAAACCTGGTGGATTGATGGCGTGCAGGAACAGGTTCGTCGTAACCGTGCGGCGA
ATCCGCCGGTGAACATTGATGCGGATCAGCTGCTGGGCATTGGCCAGAATTGGAGCACCATT
AGCCAGCAGGCGCTGATGCAGAACGAAGCGATTGAACAGGTGCGTGCGATTTGCCTGCGTGC
GTGGGAAAAAATTCAGGACCCGGGCAGCACCTGCCCGAGCTTTAACACCGTGCGTCAGGGCA
GCAAAGAACCGTATCCGGATTTTGTGGCGCGTCTGCAGGATGTGGCGCAGAAAAGCATTGCG
GATGAAAAAGCGCGTAAAGTGATTGTGGAACTGATGGCGTATGAAAACGCGAATCCGGAATG
CCAGAGCGCGATTAAACCGCTGAAAGGCAAAGTTCCGGCGGGTAGCGATGTGATTAGCGAAT
ATGTGAAAGCGTGTGATGGCATTGGCGGTGCCATGCATAAAGCCATGCTGATGGCGCAGCTC
GAGTGA

HML2-CA^{rec} protein sequence

^{1}PVTLEPMPPGEGAQEGEPPTVEARYKSFSIKMLKDMKEGVKQYGPNSPYMRTLLDSIAHGHR
LIPYDWEILAKSSLSPSQFLQFKTWWIDGVQEQVRRNRAANPPVNIDADQLLGIGQNWSTIS
QQALMQNEAIEQVRAICLRAWEKIQDPGSTCPSFNTVRQGSKEPYPDFVARLQDVAQKSIAD
EKARKVIVELMAYENANPECQSAIKPLKGKVPAGSDVISEYVKACDGIGGAMHKAMLMAQ^{246}

Supplementary Figure 1 | DNA and protein sequence of reconstructed HML2 CA^{rec}. (Upper) The codon optimised DNA sequence of the HML2 CA^{rec} synthetic gene for expression in *E. coli*. (Lower) The derived protein sequence of HML2 CA^{rec}.

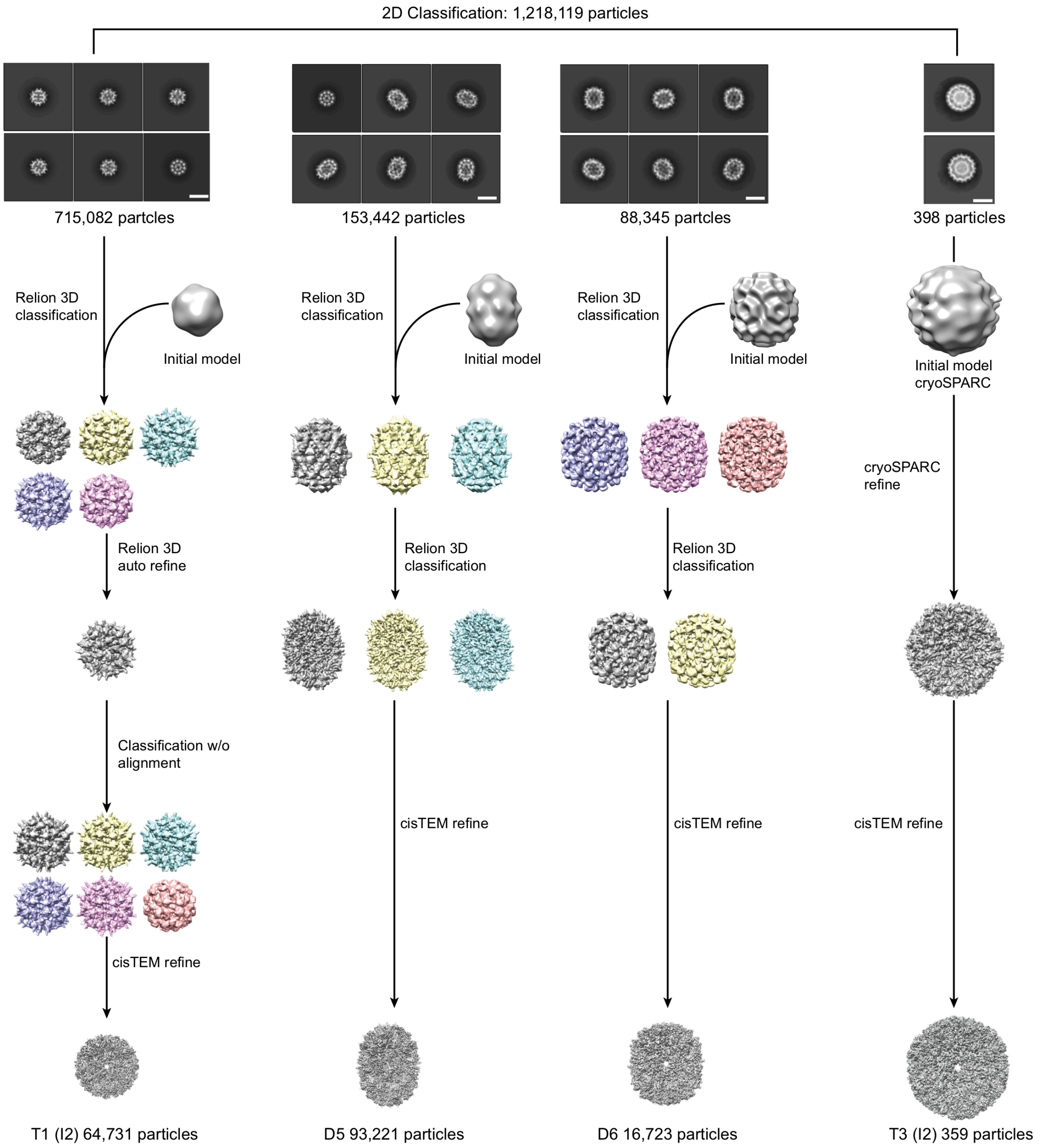

Supplementary Figure 2 | CryoEM image processing of HML2 CA^{rec} maps. Overview of the image processing and refinement procedures used to produce maps and determine HML2 CA^{rec} structures. Particle counts following 2D classification are given below the class images and below the map for final reconstruction. The software packages used at each stage are indicated and only unique steps are shown for each refinement scheme. For 2D and 3D classifications, multiple cycles of classification were employed to identify the strongest particles for refinement. 3D auto-refine in Relion was performed before refining with cisTEM.

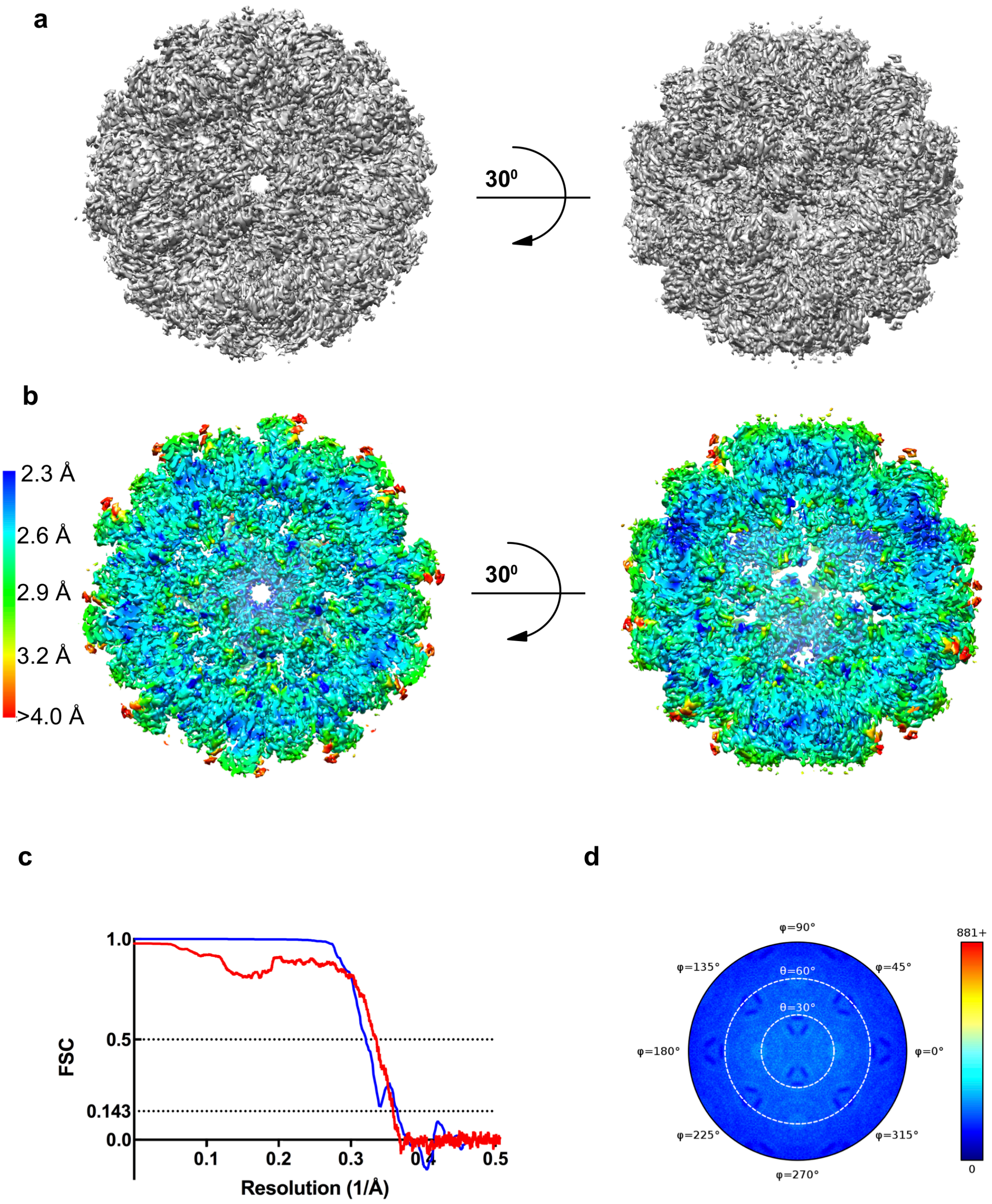

Supplementary Figure 3 | T=1 map reconstruction and resolution assessment. **a**, Views of the electron potential map, looking down a five-fold (left) and a two-fold (right) symmetry axis of the T=1 particle. **b**, Same views as in **a** but a hemisphere showing the particle interior and coloured according to local resolution values (left colour scale) as determined by ResMap. **c**, Map and map-model FSCs, the blue curve is the half-map FSC, resolution cut-off is 2.75 Å at FSC of 0.143. The red curve is the map vs model FSC calculated from final model refinement in PHENIX, (FSC of 0.5 at 3.0 Å). **d**, Particle angular distribution plot for final reconstruction. The

distribution is coloured with respect to the number of particles viewed at each angle, indicated by scale on right hand side. Calculated using cisTEM as described in Methods.

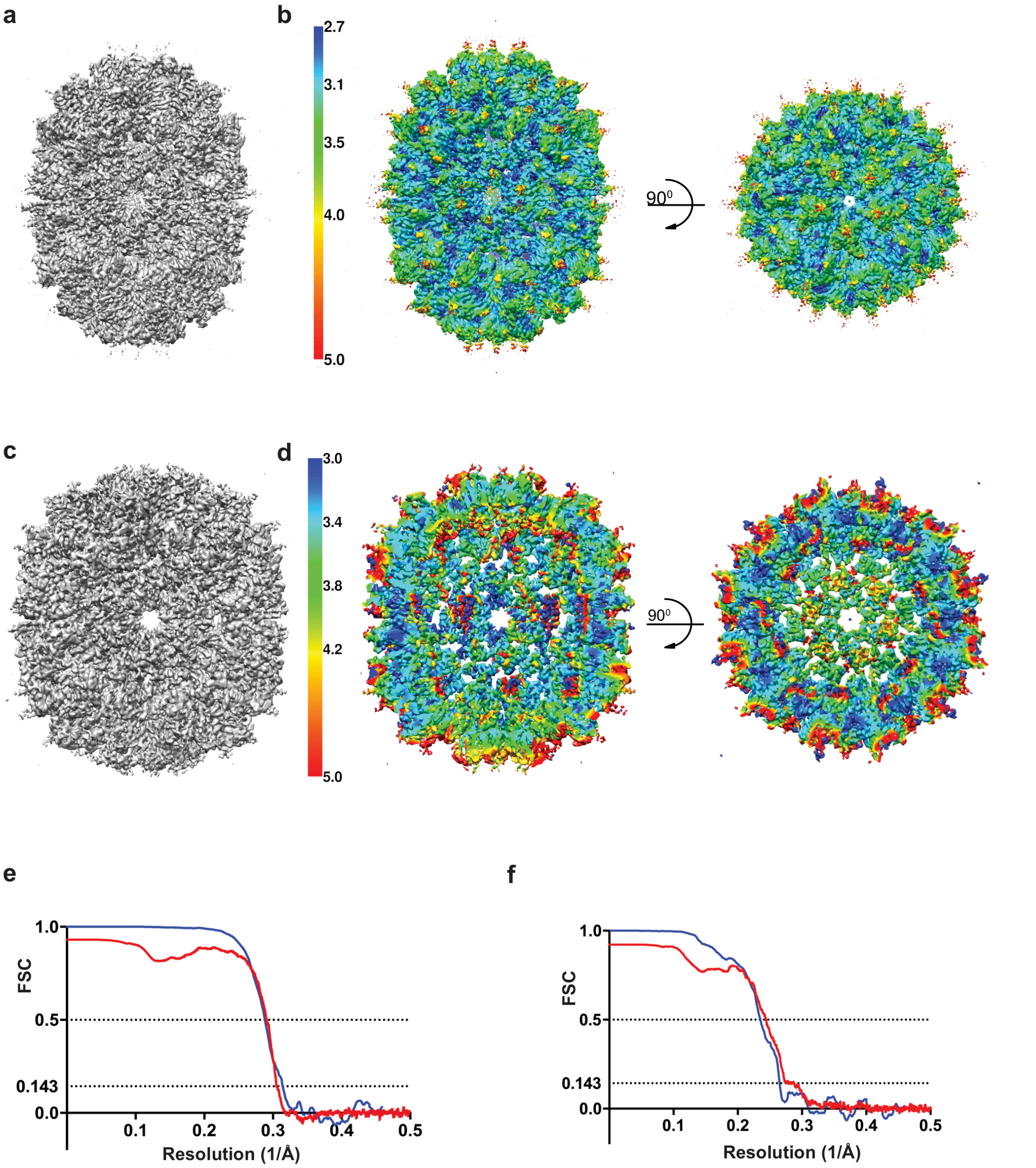

Supplementary Figure 4 | D5 and D6 map reconstruction and resolution assessment. a, Electron potential map for the D5 particle viewed along the equatorial 2-fold axis. **b**, D5 particle viewed as in **a** (left) and additionally at 90° along the 5-fold axis (right). The views are sliced to see the particle interior and coloured according to local resolution values (left colour scale) as determined by ResMap. **c**, Electron potential map for the D6 particle viewed along an equatorial 2-fold axis. **d**, D6 particle viewed as in **c** (left) and additionally at 90° along the 6-fold axis (right). Views are hemispheres to show the particle interior and coloured according to local

resolution values, determined by ResMap (left colour scale). **e** & **f**, Map and map-model FSCs, for the D5 (**e**) and D6 (**f**) particles. The blue curves are the half-map FSC, resolution cut-off is 3.18 Å and 3.77 Å for the D5 and D6 particles respectively at an FSC of 0.143. The red curves are the map vs model FSC calculated from final model refinement in PHENIX, (FSC of 0.5 at 3.4 Å and 4.1 Å for the D5 and D6 particles respectively).

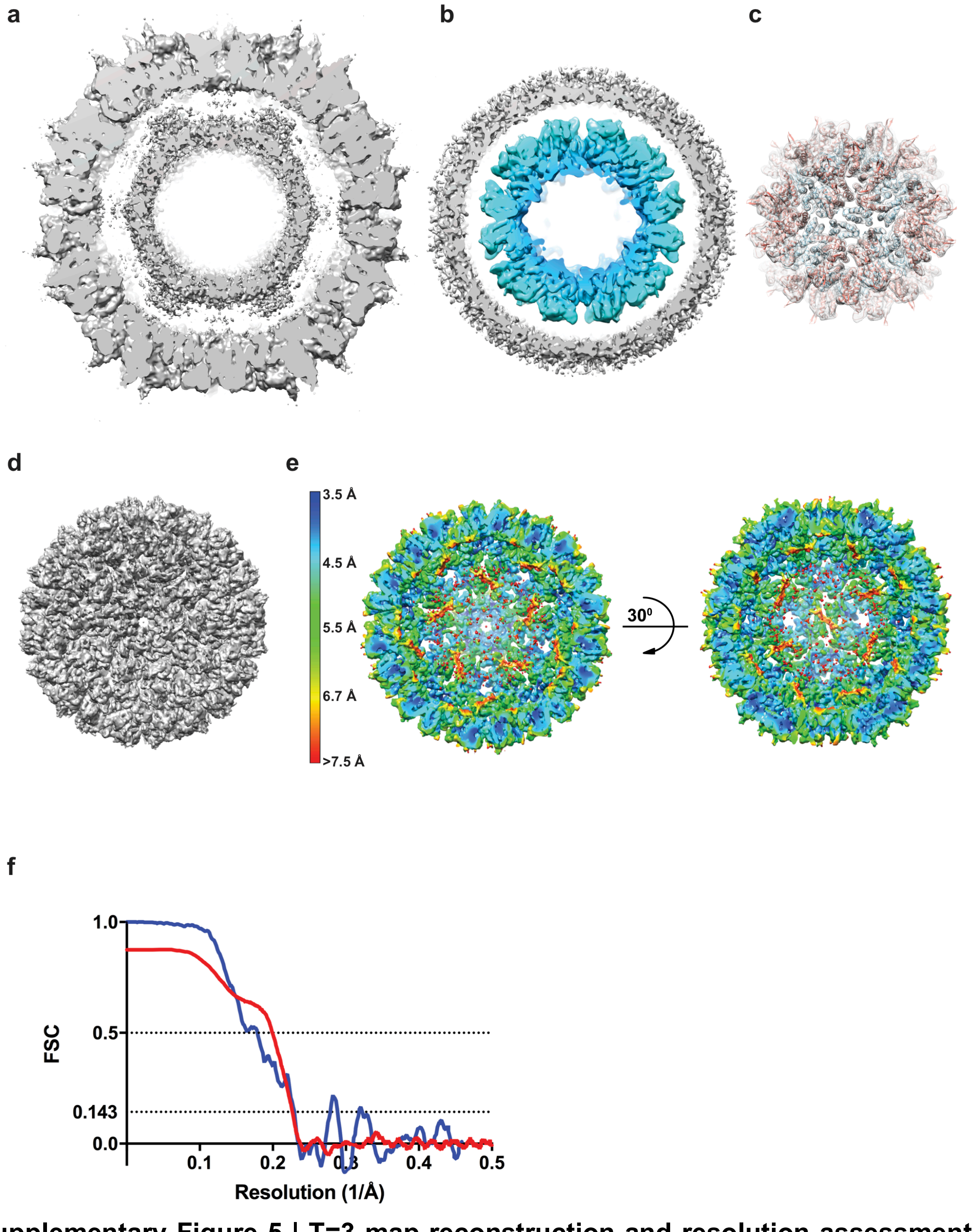

Supplementary Figure 5 | T=3 map reconstruction and resolution assessment. **a,** Section of electron potential map for T=3 particle showing a central slice through the T=3 shell revealing the additional density in the interior. **b**, Central slice of layer T=1 map (blue) resolved when taking T=3 particles and refining with mask around the central interior density. The T=3 shell (grey density) is now blurred. **c**, Docking of the high-resolution T=1 model (**Fig. 2b**) into the inner-layer T=1 map (correlation score of 0.92) of the T=3 particles. **d**, Electron potential map looking down a five-fold symmetry axis of the T=3 particle. **e**, T=3 particle viewed as in **d** (left) and

additionally along a 2-fold axis (right). The views are hemispheres to show the particle interior and coloured according to local resolution values (left colour scale) as determined by ResMap. **f**, Map and map-model FSCs. The blue curve is the half-map FSC, resolution cut-off is 4.34 Å at FSC of 0.143. The red curve is the map vs model FSC calculated from final model refinement in PHENIX, (FSC is 0.5 at 5.0 Å).

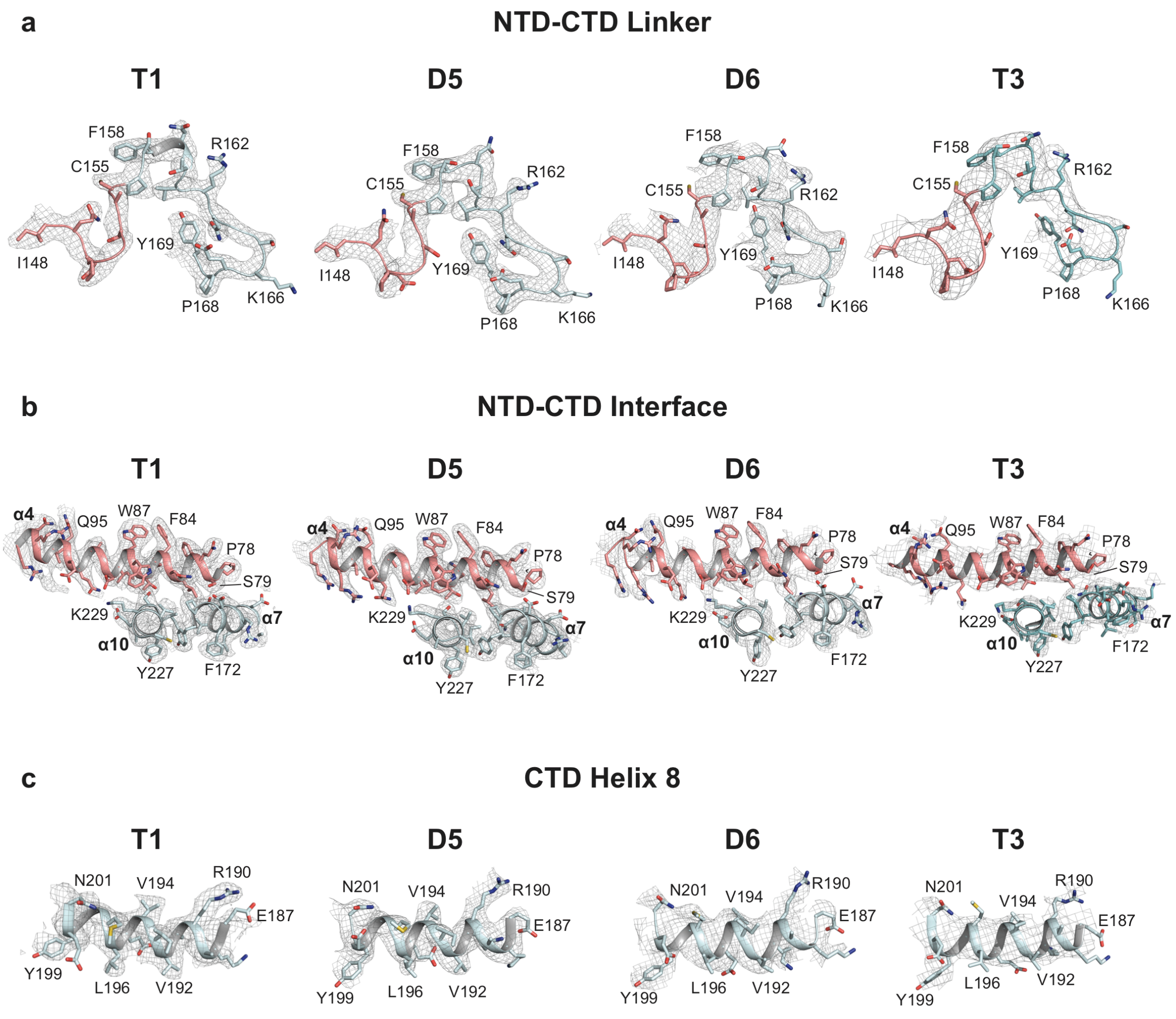

Supplementary Figure 6 | Representative cryo-EM maps and model fitting. a-c, Electron potential maps and fitted model for T=1, D5, D6 and T=3 particles for (**a**) the region around the NTD-CTD intra-domain linker (residues 148-170), (**b**) the NTD-CTD interdomain interface (NTD helix α4 and CTD helices α7, α10) and (**c**) helix α8 at the CTD-CTD interface. In all panels, maps are shown as grey mesh, the protein backbone is shown in cartoon and residues built into the density are shown in stick representation.

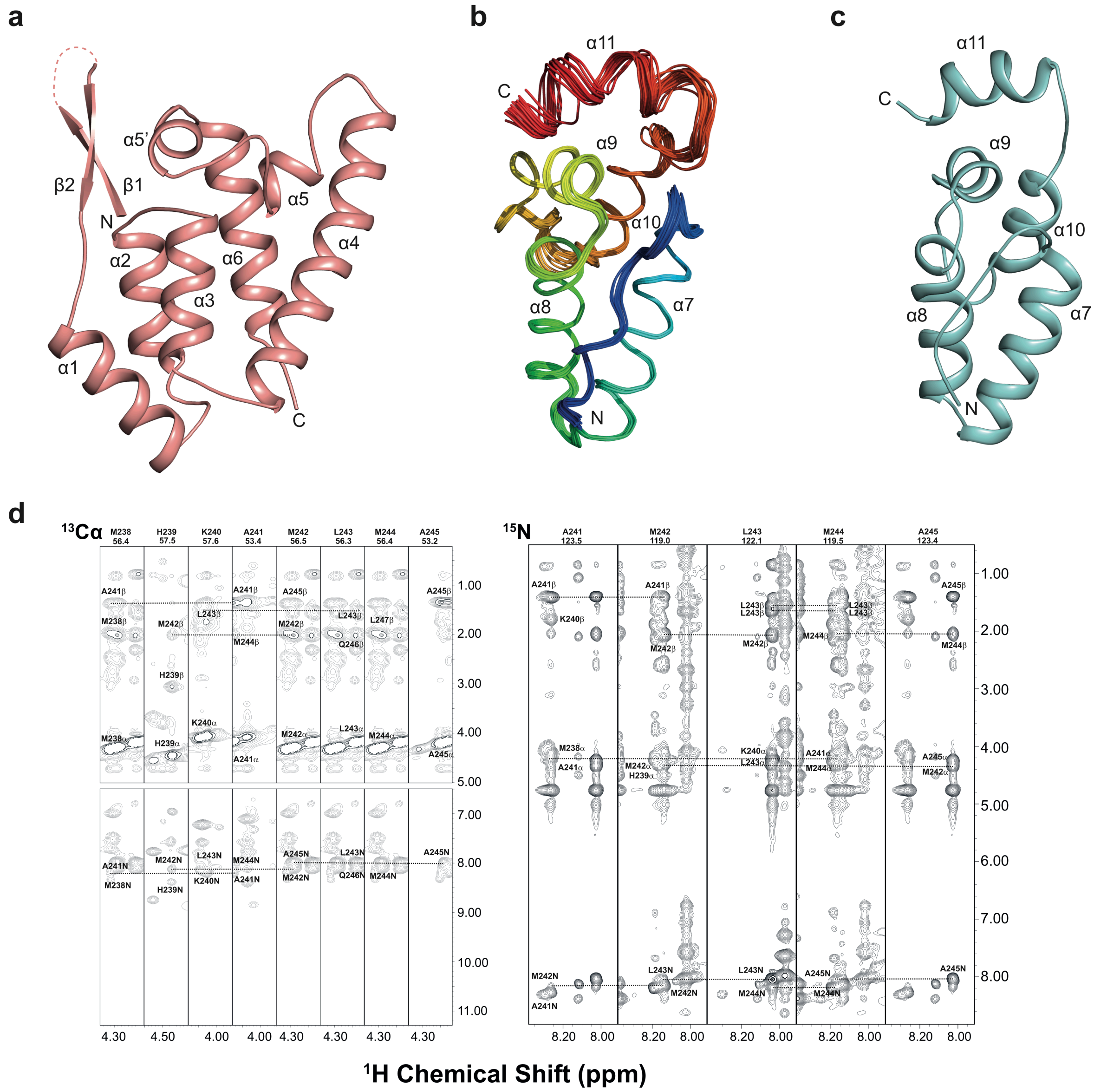

Supplementary Figure 7 | Crystal and NMR structures of HML2 CA^{rec}-NTD and CA^{rec}-CTD. a, Crystal structure of the HML2 CA^{rec}-NTD. The protein backbone is shown in cartoon representation, α-helices and β-strand secondary structure elements are labelled sequentially from the N- to the C-terminus. **b**, Family of HML2 CA^{rec}-CTD NMR structures. The protein backbone for each of the 20 conformers in the final refinement is shown in ribbon representation. The backbone is coloured from the N- to C-terminus in blue to red and α-helices are labelled sequentially. **c**, Lowest energy solution NMR structure of HML2 CA^{rec}-CTD. The backbone of the HML2 CA^{rec}-CTD monomer is shown in cartoon representation, α-helices are labelled sequentially from the N- to the C-terminus. **d**, Selected ^{1}H-^{1}H strips from the 3D ^{13}C-edited (left) and 3D ^{15}N-edited (right) HSQC-NOESY spectra. NOE cross-peaks reporting on the helical conformation of the peptide chain corresponding to helix α11

next neighbour (d_{NN}(i,i+1), $d_{\beta N}$(i,i+1)) and medium-range ($d_{\alpha N}$(i,i+3), $d_{\alpha\beta}$(i,i+3)) are highlighted using dashed lines between assigned resonances in different strips.

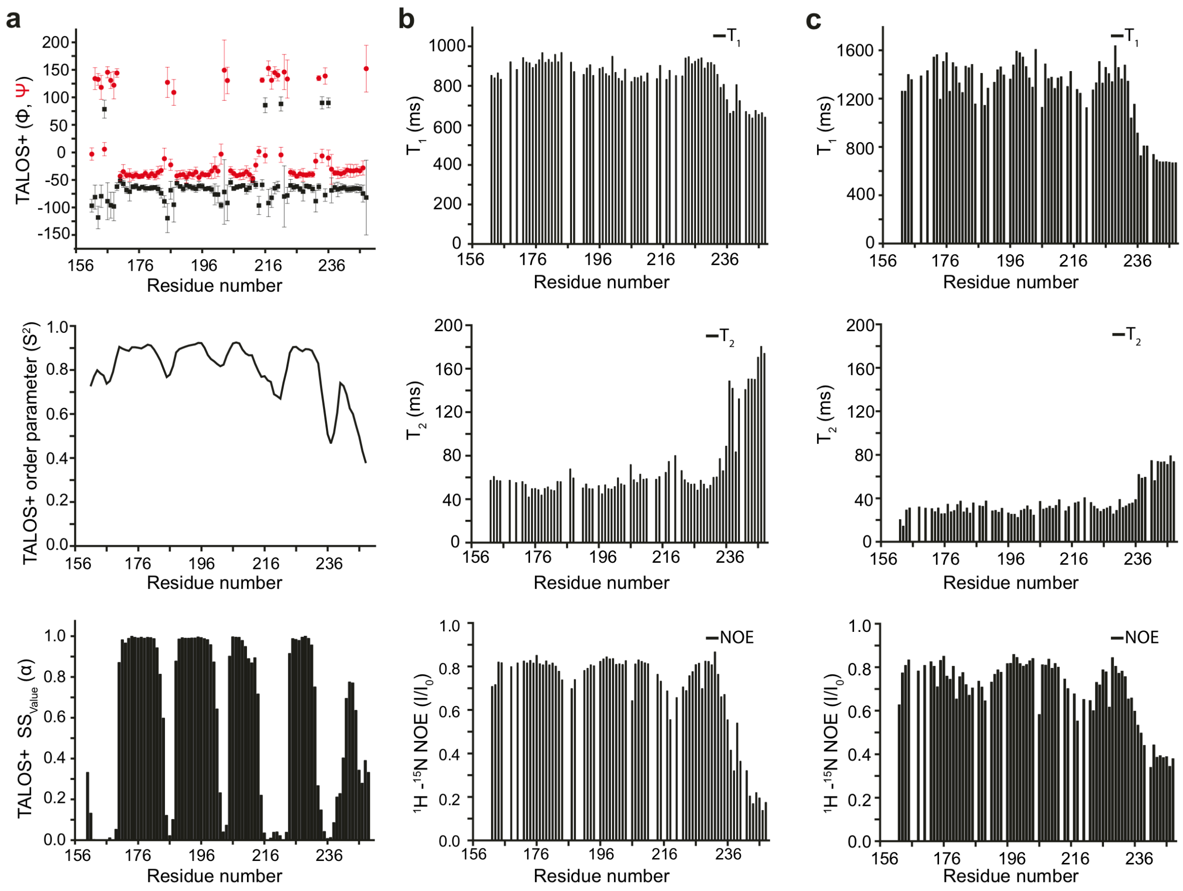

Supplementary Figure 8 | HML2 CA^{rec}-CTD NMR chemical shift and relaxation data. a, Analysis of backbone chemical shifts using TALOS+. (upper) distribution prediction of Phi (Φ, black) and Psi (Ψ, red) backbone dihedral angles, (middle) estimated ^{1}H-^{15}N bond orientational order parameter (S^2), (lower) TALOS+ assignment of α-helical secondary structure (SS). In all panels, TALOS+ parameters are plotted against sequence position, error bars represent the angular range of the standard deviation of the 10 best TALOS+ matches. **b & c**, Backbone ^{15}N relaxation parameters of HML2 CA^{rec}-CTD recorded at 25°C (**b**) and 5°C (**c**). (upper) the spin-lattice relaxation time T_1, (middle) the spin-spin relaxation time T_2 and (lower) the steady-state heteronuclear ^{1}H-^{15}N NOE recorded for each residue is plotted against sequence position. In each case, the C-terminal helix (α11; residues M238-L247) is present, but is more dynamic and has a lower order parameter than the other helices found in the CTD.

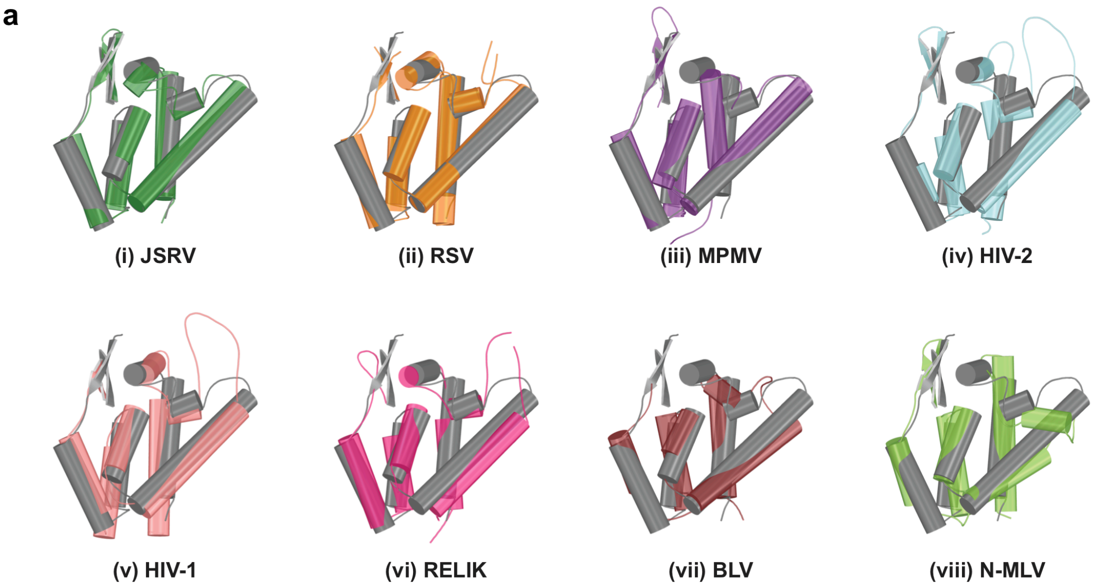

Z-score	Description	Genus	PDB ID	chain	RMSD	N-aln
10.7	JSRV CA NTD	beta	2v4x	A	1.42	127
9.0	RSV CA NTD	alpha	1em9	B	1.44	121
7.1	MPMV CA NTD	beta	2kgf	A	1.90	125
6.9	HIV-2 CA NTD	lenti	2w1v	A	2.16	114
6.6	HIV-1 CA NTD	lenti	1m9y	D	2.16	110
6.2	RELIK CA NTD	lenti	2xgu	B	2.26	114
6.1	BLV CA NTD	delta	4ph3	B	2.29	104
5.8	N-MLV CA NTD	gamma	1u7k	C	2.57	109

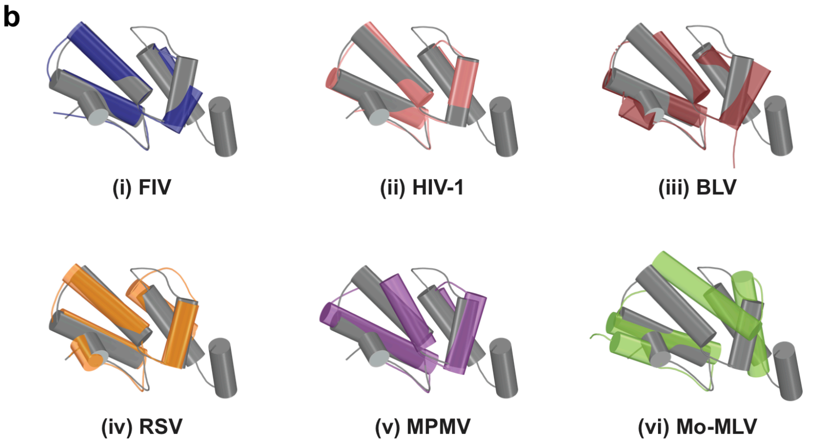

Z-score	Description	Genus	PDB ID	Chain	RMSD	N-aln
7.4	FIV CA CTD	lenti	5dck	B	1.41	68
7.3	HIV-1 CA CTD	lenti	3lry	A	1.50	70
7.3	BLV CA CTD	delta	4ph1	B	1.59	71
6.7	RSV CA CTD	alpha	3g1i	B	1.85	71
5.9	MPMV CA CTD	beta	6hwi	A	2.21	74
5.0	MoMLV CA CTD	gamma	6gza	B	2.44	72

Supplementary Figure 9 | HML2 CA^{rec} structural alignments with orthoretroviral CAs. **a**, Best-fit 3D DALI structural superimpositions of HML2 CA^{rec}-NTD with (i) JSRV CA-NTD (dark-green), (ii) RSV CA-NTD (orange), (iii) Mason-Pfizer Monkey Virus (MPMV) CA-NTD (purple), (iv) HIV-2 CA-NTD (cyan), (v) HIV-1 CA-NTD (pink), (vi) RELIK CA-NTD (magenta), (vii) Bovine Leukaemia Virus (BLV) CA-NTD

(maroon) and (viii) N-MLV CA-NTD (light-green). In all panels, molecules are shown in cartoon representation, HML2 CA^{rec}-NTD is coloured grey and α-helices are displayed as cylinders. The alignment parameters of each CA^{rec}-NTD 3D structural superposition are shown below the fits, ranked in order of decreasing Z-score. **b**, Best-fit 3D DALI structural superimpositions of HML2 CA^{rec}-CTD with (i) Feline Immunodeficiency Virus (FIV) CA-CTD (blue), (ii) HIV-1 CA-CTD (pink), (iii) BLV CA-CTD (maroon), (iv) RSV CA-CTD (orange), (v) MPMV CA-CTD (purple) and (vi) Mo-MLV CA-CTD (light-green). In all panels, molecules are shown in cartoon representation, HML2 CA^{rec}-CTD is coloured grey and α-helices are displayed as cylinders. The table below contains the alignment parameters with the fits ranked as in **a**.

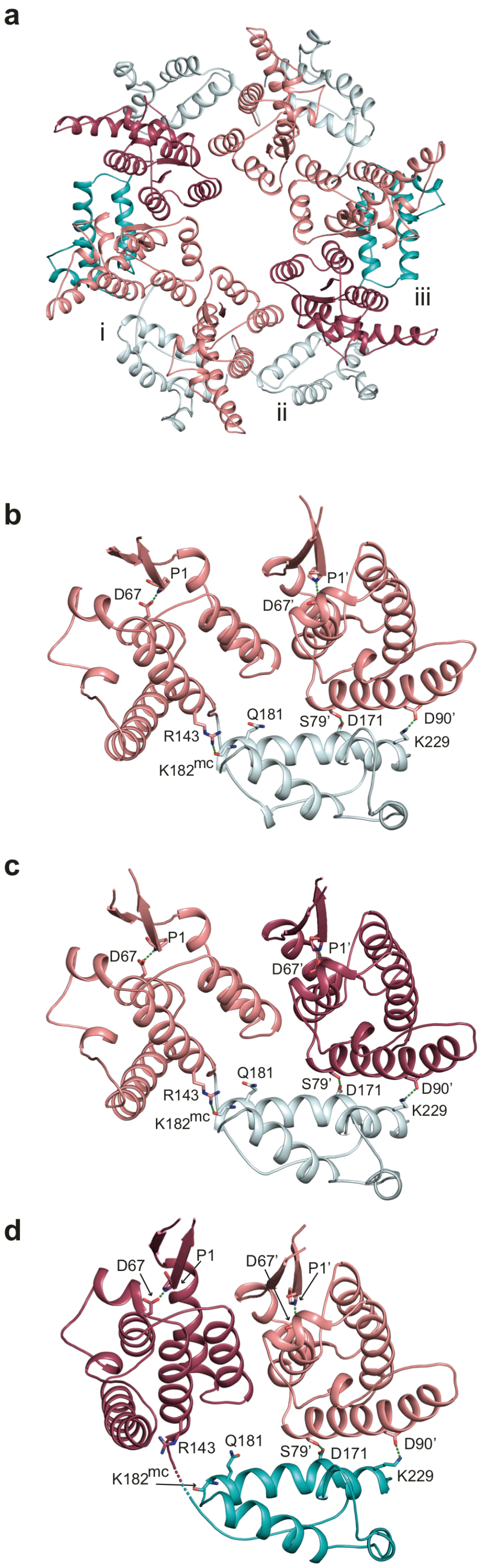

Supplementary Figure 10 | Intra-hexamer interactions in the HML2 CA^{rec} D5 particle. **a**, Cartoon representation of the D5 equatorial hexamer viewed along the pseudo six-fold symmetry axis. NTDs with CTDs that face polar pentamers or equatorial hexamers are coloured in pink and red respectively. CTDs that dimerise

with polar pentamers or equatorial hexamers are coloured in light cyan and teal respectively. The three types of non-equivalent intra-hexamer CTD-NTD interaction are indicated by i, ii and iii. **b-d**, NTD-CTD interactions in the D5 hexamer for the three types of adjacent pair, **b** Type i; CTD makes a dimer with a polar pentamer and makes a CTD-NTD interaction with an adjacent NTD whose CTD also contacts a polar pentamer. **c**, Type ii; CTD makes a dimer with polar pentamer and makes a CTD-NTD interaction with an adjacent NTD whose CTD contacts an equatorial hexamer. **d**, Type iii; CTD makes a dimer with an equatorial hexamer and makes a CTD-NTD interaction with an adjacent NTD whose CTD contacts a polar pentamer. The hydrogen bonding configuration of type i and ii resemble the T=1 pentamer, type iii resembles the D6 polar hexamer. Residues making interactions are shown as sticks with hydrogen-bonds shown as dashes, the prime (’) notation indicates the adjacent NTD and “mc” indicates a main-chain interaction. The conserved N-terminal P1 and α3-D67 interaction is also shown in each NTD for orientation.

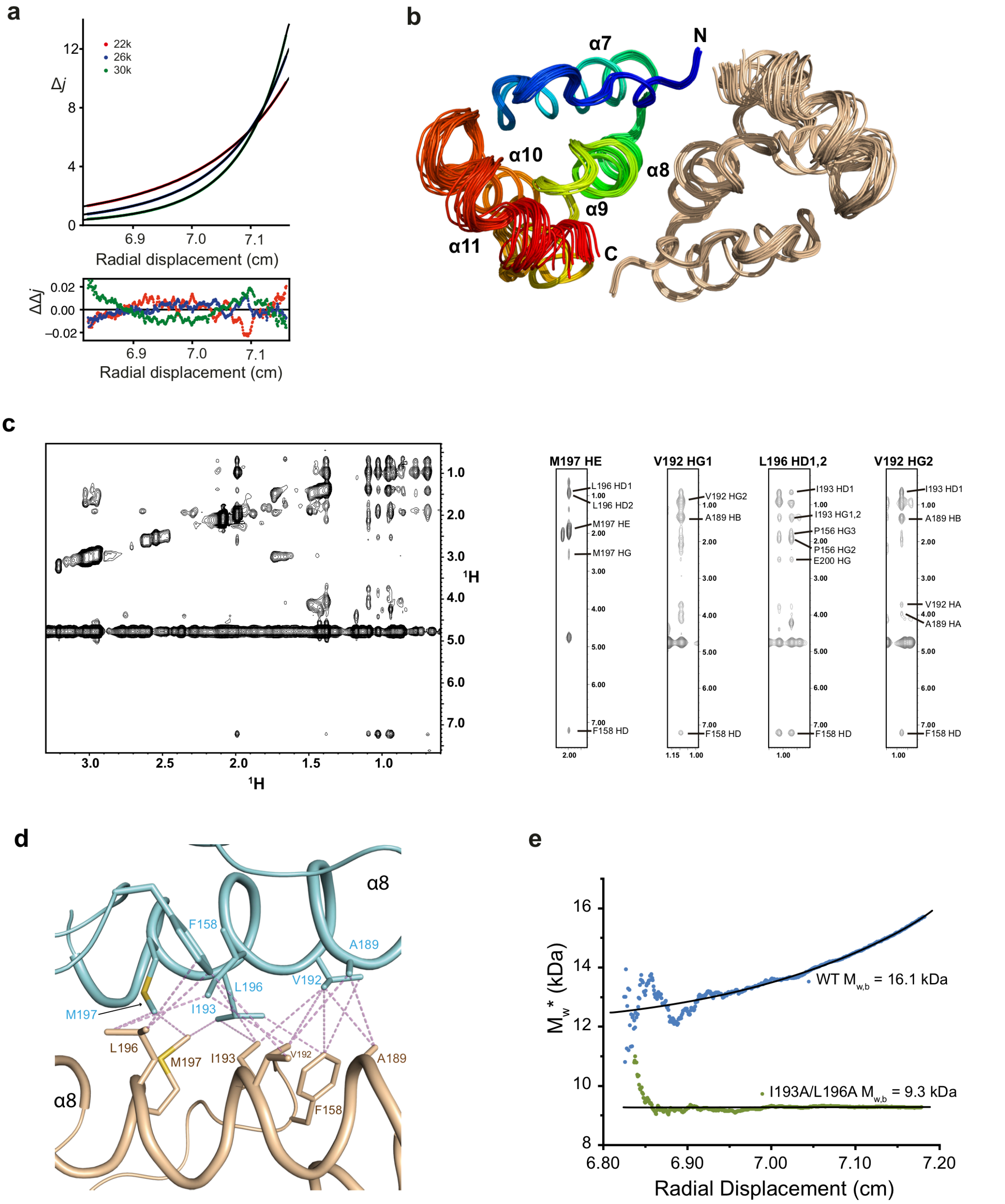

Supplementary Figure 11 | HML2 CA^{rec}-CTD dimer. a, Multispeed sedimentation equilibrium profile determined from interference data collected on 200 μM HML2 CA^{rec}-CTD. Data was recorded at the speeds indicated. The solid lines represent the global best fit to the data with a monomer-dimer equilibrium model, the lower panel shows the residuals to the fit. **b**, Family of HML2 CA^{rec}-CTD homodimer NMR structures. The protein backbone for each of the 20 conformers in the final refinement is shown in ribbon representation. The backbone of one monomer is coloured from the N- to C-terminus in blue to red, α-helices are labelled sequentially.

The other monomer is shown in wheat. **c**, (Left) 2D projection of a region of the HML2 CA^{rec}-CTD 3D F1-$^{13}C/^{15}N$-filtered, F3-^{13}C-edited NOESY-HSQC spectrum showing intermolecular NOE correlations. (Right) selected ^{1}H-^{1}H strips from the filtered spectrum. Intermolecular NOE correlations from residues at the dimer interface are indicated. **d**, Mapping of intermolecular CTD-CTD NOEs. A detailed view of the CTD-CTD dimer interface is displayed with the protein backbone shown in cartoon representation, CTD monomers are coloured in cyan and wheat. Aliphatic residues with assigned intermolecular NOEs (F158, A189, V192, I193, L196 and M197) are labelled and shown in stick representation. The purple dashes between atoms represent the observed strong inter-monomer NOEs. **e**, Sedimentation equilibrium M* analysis of HML2 CA^{rec}-CTD and CA^{rec} CA-CTD(I193/L196) mutant. The point average molecular weight (M_w*) is plotted against radial position for CA^{rec}-CTD (blue) and CA-CTD(I193/L196) (green). The lines are the fit to the M* transformation of the C(M) function to yield the extrapolated weight-averaged molecular mass at the cell bottom ($M_{w,b}$). Analysis of the mutant yields the monomer molecular mass and shows no indication of self-association.
