# Supplementary material for: Structural basis for Fullerene geometry in a human endogenous retrovirus capsid
Source: Nat Commun. 2019 Dec 20;10:5822. doi: 10.1038/s41467-019-13786-y (PMC6925226; doi:10.1038/s41467-019-13786-y)
Supplement: Supplementary file 2 — Description of Additional Supplementary Files [file 41467_2019_13786_MOESM2_ESM.pdf]

Description of Additional Supplementary Files

File name: Supplementary Movie 1

Description: Hexamer to pentamer transformation. Movie comparing the structure of the D6 polar hexamer and the T=1 pentamer. The movie highlights the changes in monomer packing when a different number of CA monomers are accommodated in the ring. NTDs are coloured salmon, CTDs are coloured cyan. Morph was created using Chimera.
